# Pivotal role of O-antigenic polysaccharide display in the sensitivity against phage tail-like particles in environmental *Pseudomonas* kin competition

**DOI:** 10.1038/s41396-022-01217-8

**Published:** 2022-03-10

**Authors:** Clara Margot Heiman, Monika Maurhofer, Sandra Calderon, Mélanie Dupasquier, Julien Marquis, Christoph Keel, Jordan Vacheron

**Affiliations:** 1 Department of Fundamental Microbiology, University of Lausanne, Lausanne, Switzerland; 2 Plant Pathology, Institute of Integrative Biology, Swiss Federal Institute of Technology (ETH) Zurich, Zurich, Switzerland; 3 Lausanne Genomic Technologies Facility, University of Lausanne, Lausanne, Switzerland

**Keywords:** Bacteriophages, Environmental microbiology

## Abstract

Environmental pseudomonads colonize various niches including insect and plant environments. When invading these environments, bacteria are confronted with the resident microbiota. To oppose with closely related strains, they rely on narrow-spectrum weaponry such as tailocins, i.e., phage tail-like particles. Little is known about the receptors for these tailocins especially among phylogenetically closely related species. Here, we studied the interaction between an R-tailocin from *Pseudomonas protegens* CHA0 and a targeted kin, *Pseudomonas protegens* Pf-5. Using genome-wide transposon insertion sequencing, we identified that lipopolysaccharides are involved in the sensitivity of Pf-5 towards the tailocin of CHA0. By generating Pf-5 lipopolysaccharide mutants and exposing them to extracted tailocin, we specified the two O-antigenic polysaccharides (O-PS) targeted by the tailocin. We affirmed the role of these O-PS through competition assays in vitro as well as in insects. Further, we demonstrate that O-PS are double-edge swords that are responsible for the sensitivity of *P. protegens* towards tailocins and phages produced by their kin, but shield bacteria from the immune system of the insect. Our results shed light on the trade-off that bacteria are confronted with, where specific O-PS decorations can both be of benefit or disadvantage depending on the host environment and its bacterial inhabitants.

## Introduction

*Pseudomonas protegens* bacteria are found in very diverse environmental niches and notably are known for their ability to colonize two different eukaryotic hosts, plants and insects. Indeed, several strains are well-known root colonizers with plant-beneficial activities, in addition to being invaders of certain plant pest insects, exerting entomopathogenic activities or exploiting their insect host as vector for dispersal [1–5]. To colonize these contrasting hosts, *P. protegens* shows an outstanding capacity to establish itself efficiently within the resident host microbiota. For insect invasion, these bacteria deploy a vast arsenal of weapons allowing them to face the gut microbiota [5, 6]. For instance, the expression of genes involved in the production of broad-spectrum antimicrobial compounds increased following the ingestion of *Pseudomonas protegens* type strain [7, 8] and widely used model environmental pseudomonad CHA0 (hereafter CHA0) by the pest insect *Plutella xylostella* [6, 9]. Following the ingestion by another pest insect, *Pieris brassicae*, CHA0 used its type VI secretion system to ward off *Enterobacter* sp. dwelling on the gut epithelial barrier to cross through to and colonize the hemolymph [10]. Once inside the blood system of the insect, these *Pseudomonas* can escape the insect immune defense by displaying a specific lipopolysaccharide (LPS) at their cell surface [11]. As they are hidden from the immune system, they can multiply and start producing the entomotoxin Fit leading to a systemic infection causing the death of the insect [6, 12–15].

However, CHA0 may not only be confronted with phylogenetically distant bacteria but is likely to encounter other closely related *Pseudomonas* that would exploit the insect niche. Close relatives are generally resistant to the broad-spectrum weaponry since they share highly similar, if not almost identical, genetic backgrounds [16–18]. Nonetheless, *Pseudomonas* strains also deploy weapons that are very specific, even down to the strain level, notably tailocins, i.e. highly specialized phage tail-like particles [18–26]. Tailocins are antimicrobial structures that are thought to be evolved from bacteriophages belonging to the *Caudoviridales* order [21, 23, 27–29], with two main types, R-tailocins (rigid and contractile) and F-tailocins (flexible and non-contractile) [27].

Following release by explosive cell lysis [18], tailocins will reach and bind to their target bacteria by attaching to specific cell-surface receptors similarly to bacteriophages [21–23, 30, 31]. Receptor-binding proteins such as tail fibers, tail spikes or the tail tip are used by the tailocin structure to recognize the target receptor [25, 32]. For R-tailocins, the contact between the receptor-binding protein and their specific receptor will induce the contraction of the tail sheath to push the internal rigid tail tube-spike complex toward the membrane of the target, disrupting it and leading to the death of the targeted bacterial cell [23, 31–34].

Previously, we showed that the CHA0 genome encompasses prophage-like clusters encoding two R-tailocins (tailocin #1 and tailocin #2) as well as a myovirus and a siphovirus [18]. The tailocin #2 is present in most *Pseudomonas* strains investigated so far, whereas the tailocin #1 is harbored by a few strains and displays a highly specialized spectrum against specific strains belonging to the *Pseudomonas protegens sensu strico* species including *P. protegens* Pf-5 (hereafter Pf-5, [18, 28]). Although the killing activity of tailocin #1 of CHA0 was demonstrated, its receptors on Pf-5 are unknown [18].

A number of studies demonstrate that LPS are involved in the sensitivity of a target bacterium towards R-tailocins [23, 25, 35]. LPS are formed out of three major parts: the lipid A, a hydrophobic structure that anchors the LPS into the outer membrane, the core oligosaccharide, a highly phosphorylated negatively charged structure that provides membrane stability, and finally an O-antigenic polysaccharide (O-PS, also termed O-antigen), which is the exposed part of the LPS [36]. Generally, the lipid A and core oligosaccharide form the conserved part of the structure, while the O-PS is highly variable and differs between strains [36]. Moreover, some strains expose multiple different types of O-PS on their cell surface [11, 36, 37]. The O-PS is synthesized and can be transported separately from the lipid A core oligosaccharide [36]. As they can be transported separately, some LPS, called uncapped, lack the O-PS [11, 36, 37]. LPS formed out of all three parts are called capped [11, 36, 37]. Although the LPS of CHA0 are known to be composed of a short (OSA, O-specific antigen) and a long (OBC3, O-PS biosynthesis cluster 3) O-PS structure, the LPS profile of Pf-5 is poorly documented [11].

The interaction between CHA0 and Pf-5 is of interest as these strains have very high genomic similarity. We suggest that the arms race to colonize the same niches between these two kin bacteria does not entirely rely on weapons to attack their adversary but also defense systems against the assault of their opponent. In this study, through a genome-wide transposon insertion sequencing (Tn-seq) approach, we scanned for candidate genes involved in the resistance and sensitivity of Pf-5 against a highly specialized phage tail-like weapon of CHA0, *i.e*., tailocin #1. Subsequently, we used a reverse genetic approach to evaluate the importance of these genes during dual competition in vitro and during host colonization (*in insecta* and *in planta*). Through these experimental approaches, we have identified that LPS of Pf-5 are key components in its immunity against the R-tailocins of CHA0 but are also imperative to evade the immune system of the insect. These findings support the hypothesis that cell surface decorations, more specifically LPS, are double-edge swords that can both be of disadvantage or benefit for the bacterium when it faces the viral weaponry of adversaries or the immune defense of the eukaryotic host.

## Material and methods

### Bacterial strains, plasmids, media, and culture conditions

Strains and plasmids used in this study are listed in Supplementary Tables 1, 2 and 3. *Pseudomonas* strains were routinely cultured on nutrient agar (NA) and in nutrient yeast broth (NYB) at 25 °C. *Escherichia coli* was grown in lysogeny broth (LB) at 37 °C. When required media were supplemented with selective antibiotics at the following concentrations: ampicillin, 100 µg/mL; chloramphenicol, 50 µg/mL; gentamicin, 10 µg/mL; kanamycin, 25 µg/mL.

### Extraction of phage(-related) particles

*Pseudomonas* strains (Supplementary Table 1) were restarted into fresh NYB from an overnight culture. To induce the production of tailocins and phage particles, 3 μg/mL of mitomycin C was added when the cultures reached exponential growth phase (optical density [OD] at 600 nm of 0.3–0.4), followed by an incubation at 25 °C for a minimum of 3 h. Induced cultures were then centrifuged at 6000 rpm for 15 min. The phage(-related) particles present in the supernatant (i.e., tailocins and/or phages) were caught onto Amicon Ultra-15 centrifugal filters with a molecular weight cut-off of 50 kDa (Millipore). Particles were resuspended from the filters by using 1 mL of Tn50 buffer. Extracts of individual tailocins were obtained from mutants of CHA0 that are defective for the other phage(-related) particles (Supplementary Table 2) as detailed in [18].

### Transposon mutant library construction

An overnight culture of *P. protegens* Pf-5 was grown at 35 °C, centrifuged and washed twice with a MOPS glycerol solution to obtain a suspension of competent cells. These cells were electroporated with the plasmid pRL27 containing the mini-Tn5 transposon [38] and immediately rescued in 1 mL of Super Optimal broth with Catabolite repression medium (SOC) for 2.5 h at 35 °C. The cell suspensions then were serially diluted and plated onto NA plates with 25 µg/mL of kanamycin. Following incubation for 24 h at 25 °C, the totality of the Km-resistant colonies from a selection of 10 NA plates per strain, yielding a total of approximatively 11 million colonies, were harvested into sterile 0.8% NaCl-solution, homogenized and centrifuged to concentrate the full Tn5-library into a final volume of 5 mL. Aliquots of 1 mL were stored with glycerol at −80 °C for subsequent use.

### Exposure of the *P. protegens* Tn-mutant library to tailocins and Tn-seq analysis

Aliquots of 150 µL of the Tn-mutant library were added to 8 mL of NYB and incubated for 8 h at 25 °C. The OD at 600 nm of these cultures was adjusted to 0.01 (corresponding to approximately 2 × 10^6^ cells/mL) in new tubes containing fresh NYB. The Tn-mutant library was exposed to 400 µL of a purified suspension of the tailocin #1 of CHA0, containing approximately 200000 tailocin particles/mL (based on a semi-quantification approach [18]). Three independent replicates were performed including each time a control condition without the tailocins. After 10 h of incubation at 25 °C, the cells were centrifuged and washed twice with sterile H_2_O to remove any traces of extracellular DNA from lysed cells in the supernatant. Genomic DNA was then extracted from the cell pellets using the MagAttract HMW DNA kit (Qiagen). The Tn-seq DNA library preparation, Illumina sequencing, sequence processing and statistical analysis was performed as detailed in the Supplementary Information.

### Phylogenetic analysis and comparison of genomes

The *P. protegens* (Pp)/*P. chlororaphis* (Pc) phylogenetic tree was based on the concatenation of the housekeeping genes *rpoD*, *rpoB* and *gyrB*. Nucleotide sequences were retrieved from the genome sequences (Supplementary Table 1) using BLASTn (https://blast.ncbi.nlm.nih.gov/Blast.cgi). The sequences were aligned using MUSCLE [39], concatenated and used to build a Maximum Likelihood tree with PhyML [40]. The genome comparisons of CHA0 and Pf-5 were performed on the MaGe platform (https://mage.genoscope.cns.fr/).

### Lipopolysaccharide and O-antigenic polysaccharide cluster identification

The gene clusters encoding core-LPS and O-PS formation in Pf-5 were identified by performing BLASTn and BLASTp analyses (targeting, e.g., nucleotide sugar biosynthesis proteins, glycosyltransferases, LPS transporters) based on the well-characterized core-LPS and O-PS clusters in the genome of CHA0 [11] using the NCBI platform (https://blast.ncbi.nlm.nih.gov) with a minimum of 70% nucleotide sequence identity over 70% of the coding sequence.

### Mutant construction

To test the role of candidate genes in tailocin resistance, they were deleted in Pf-5 according to the results obtained in the Tn-seq experiment. Deletion mutants (Supplementary Table 2) were constructed using the suicide vector pEMG and the I-SceI system [41] with a protocol adapted for *P. protegens* [10, 18], with plasmids and primers listed in the Supplementary Tables 3 and 4.

### Lipopolysaccharide extraction and characterization

LPS were extracted from Pf-5 and its LPS mutants following previously established protocols [11, 42]. The samples were migrated with a sodium dodecyl sulfate polyacrylamide gel electrophoresis (SDS-PAGE) directly after the proteinase K treatment using either an 8% or 12% acrylamide gel and visualized by silver staining. The PageRuler or the PageRuler Plus Prestained Protein Ladders (Thermo Scientific) were used as a molecular mass standards.

### Assays for sensitivity of *P. protegens* to phage(-related) particles

The sensitivity of CHA0 and Pf-5 and their mutants towards CHA0 tailocins and phage particles extracted from different Pp/Pc subgroup strains was assessed by spotting the phage(-related) particle extracts (containing tailocins and phages) on lawns of the different bacterial strains and assessing the formation of lytic zones as previously described [18].

### Bacterial competition assays in vitro and in vivo

To test the competitiveness of Pf-5 derivatives lacking specific receptors for CHA0 tailocin, we tested the Pf-5 wild-type and LPS mutants in pairwise competition against wild-type CHA0 or tailocin mutant derivatives in liquid medium (in vitro), in insecta and in planta as detailed in Supplementary information.

### Statistics and reproducibility

All experiments were performed using at least four biological replicates with each at least three technical replicates as detailed in the figure legends. Data were analyzed using R studio version 4.0.3 and considered significantly different when *P* < 0.05. The data were verified and transformed for normal distribution and variance homogeneity using Shapiro-Wilk tests and Bartlett tests, respectively. ANOVA coupled with HSD-Tukey test were performed. When the normal distribution was not respected, non-parametric tests were performed such as Wilcoxon tests or Kruskal-Wallis tests applying a Bonferroni correction were used to assess significant differences between conditions.

## Results

### The genomic differences between CHA0 and its kin Pf-5 account for the differences in phage tail-like particle sensitivity patterns

**Fig. 1:**
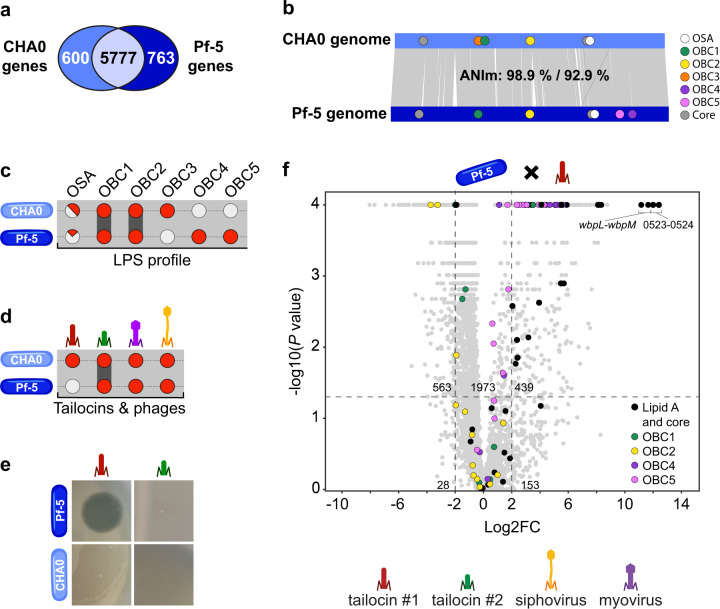
The O-polysaccharide (O-PS) gene clusters that differ between the highly similar *Pseudomonas protegens* strains CHA0 and Pf-5 account for the sensitivity towards the tailocin #1 of CHA0. **a** Venn diagram showing the genes in common (core genome; in light blue) and the genes unique to each strain (CHA0 in blue and Pf-5 in dark blue). **b** Genome alignment of CHA0 (blue, top) and Pf-5 (dark blue, bottom) with Average Nucleotide Identity (ANI) analysis results. OBC O-PS biosynthesis cluster, OSA O-specific antigen. **c** LPS composition of both strains. Grey circles represent absence, while red circles represent presence. Dark grey connectors show conservation between both strains **d** tailocins and phages found within the genomes of both strains. Grey circles represent absence, while red circles represent presence. **e** Tailocin #1 and tailocin #2 sensitivity profile of Pf-5 and CHA0. **f** Transposon sequencing results for the sensitivity of Pf-5 towards tailocin #1. Vertical dotted lines show the log Fold-Change (FC) cut-off and the horizontal dotted lines correspond to a significant *p* value.

CHA0 and Pf-5 are very closely related strains that are part of the same species, the *Pseudomonas protegens sensu stricto* [18]. Indeed, these two strains have 5777 genes in common out of approximately 6500 genes (Fig. 1a, b, Dataset 1). These shared genes display 98.9% of identity (ANIm calculation). In the diverging genes, some were found to encode cell surface elements, notably LPS. Even though Pf-5 and CHA0 share the conserved lipid A core oligosaccharide, there are some differences in their OBC clusters encoding different exposed O-PS structures. CHA0 and Pf-5 share the OBC1 and OBC2, but possess O-PS clusters that the other strain does not. CHA0 harbors an incomplete OSA cluster (compared to the prototype *P. aeruginosa* OSA cluster) and an OBC3 [11], while Pf-5 carries OBC4 and OBC5, and its OSA cluster is further reduced (Fig. 1c, Supplementary Figure 1, [11]). Further genetic differences were found in the clusters encoding phage(-related) particles (i.e., tailocins and temperate phages) (Fig. 1d). Pf-5 harbors the tailocin #2 also present in CHA0, but not the tailocin #1 present in CHA0 to which it is sensitive (Fig. 1d, e). Moreover, the *Myoviridae* and *Siphoviridae* prophages differ between both strains. Both strains are resistant to their own phage(-related) particles (Fig. 1e, [18]).

We used a Tn-seq approach to identify the molecular determinants involved in the sensitivity of Pf-5 towards the tailocin #1 of CHA0. We generated a Tn-mutant library where the transposon was inserted in 96% of Pf-5 genes in the control condition (Supplementary Table 5). Following exposure of the Tn-mutant library to the tailocin #1 of CHA0, we identified several LPS-related candidate genes for the sensitivity of Pf-5 towards the phage tail-like particles, notably those of the OBC4 and OBC5 clusters (Fig. 1f, Dataset 2). Several genes associated with the lipid A-core oligosaccharide also appear to be involved in the tailocin sensitivity of Pf-5 such as *wbpL*, *wbpM* and PFL_0523-0524 (Fig. 1f). They are required for the correct assembly of the LPS core and, without them, O-PS structures cannot bind to the core resulting in uncapped profiles.

Thus, although CHA0 and Pf-5 are very closely related genetically, they have differences in the LPS profiles that could account for different sensitivity patterns towards the tailocins of CHA0.

### The OBC4 and OBC5 clusters are involved in the sensitivity of Pf-5 towards the tailocin #1 of CHA0 in vitro

As some of the candidate genes following the Tn-seq analysis involved in the sensitivity of Pf-5 are part of LPS gene clusters, we used reverse genetics to investigate the LPS profile of Pf-5. In addition to deleting the entire four O-PS clusters (OBC1, OBC2, OBC4, OBC5), we also removed specific genes within these clusters as well as in the OSA and lipid A-core clusters known to be important for the correct assembly of the different LPS structures (Supplementary Figure 1, Supplementary Table 2).

**Fig. 2:**
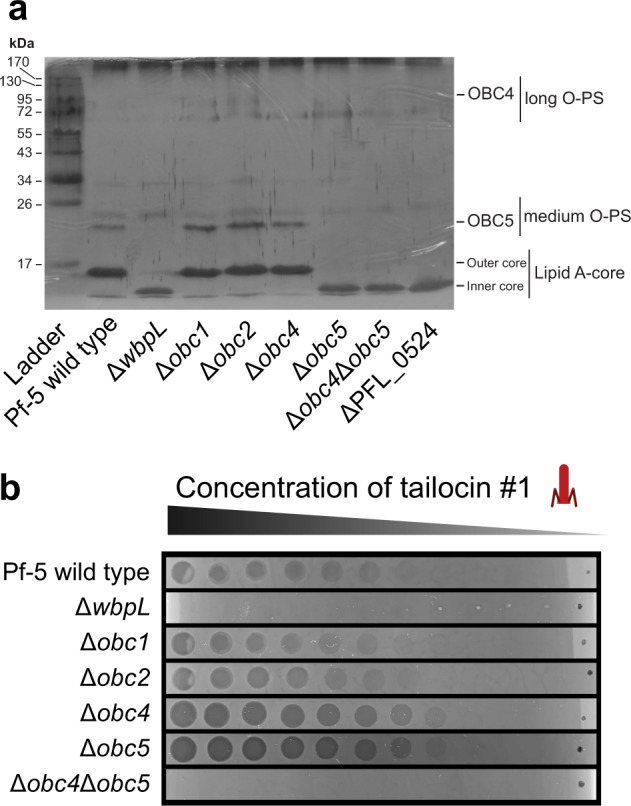
Removal of the major O-polysaccharides OBC4 and OBC5 makes P. protegens Pf-5 resistant to the tailocin #1 of P. protegens CHA0. **a** LPS profile of Pf-5 wild-type, ΔwbpL, ΔPFL_0524 and entire O-PS cluster deletion mutants. SDS-PAGE on LPS extracts was performed using a 12% acrylamide gel and components were visualized by silver staining. **b** Bacterial lawns of the different Pf-5 LPS mutants were exposed to extracts of the tailocin #1 of CHA0 that were serially diluted at a ratio of 1:4. As controls, Pf-5 and derivatives were also exposed to extracts containing all the phage(-related) particles extracted from CHA0, one with only the tailocin #2 and one void of any particles (Supplementary Figure 3).

Subsequently, we extracted the LPS from these different mutants and visualized the different patterns on polyacrylamide gels (Fig. 2a). The wild-type Pf-5 has a LPS pattern encompassing medium and long O-PS (Fig. 2a). As in CHA0, the deletion of *wbpL* encoding the initial glycosyltransferase for O-PS resulted in an non-functional core, producing a downshift of the banding pattern and the absence of medium and long O-PS (Fig. 2a, Supplementary Figure 2b, [11]). We also deleted the gene PFL_0524, which codes for a glycosyltransferase involved in the biosynthesis of the lipid A-core [43], producing a similar phenotype lacking O-PS structures (Fig. 2a). The defective cores of both these mutants make them unable to bind O-PS structures, and thus they exhibit uncapped LPS profiles. The deletion of either OBC1 (Δ*obc1*) or OBC2 (Δ*obc2*) had no influence on the LPS profile, as the banding pattern remained the same as for wild-type Pf-5 (Fig. 2a). Conversely, OBC4 and OBC5 appear to specify the most prevalent O-PS types present on the cell surface of Pf-5. Indeed, the deletion of the entire OBC4 (Δ*obc4*) caused a downshift of the banding pattern with the loss of the heaviest band (Fig. 2a), suggesting that his cluster encodes one long O-PS. Moreover, when the entire OBC5 was deleted (Δ*obc5*), the band at about 20 kDa was no longer visible, indicating that the OBC5 produces a medium length O-PS (Fig. 2a). Interestingly, the banding pattern of Δ*obc5* also showed the loss of the band at 15 kDa corresponding to the outer core, suggesting that the OBC5 encodes proteins involved in the core formation. To further understand the LPS profile of Pf-5, individual mutants were constructed within the OBC4 and OBC5 (Supplementary Figure 1, Supplementary Table 2). Interestingly, some genes specific to each one of these clusters have an effect on the banding pattern associated with the other O-PS gene cluster (Supplementary Figure 2). When both OBC4 and OBC5 were deleted (Δ*obc4*Δ*obc5*), the banding pattern was the same as for Δ*wbpL* (Fig. 2a). These results highlight that the LPS profile of Pf-5 is composed mostly of OBC4 and OBC5 O-PS (Fig. 2a).

To specifically identify which O-PS structure is the receptor for the tailocin #1, *i.e*., to which Pf-5 is sensitive (Fig. 2b), we exposed deletion mutants of these different O-PS clusters to purified tailocin #1 of CHA0. The Pf-5 Δ*wbpL* mutant was resistant to all phage(-related) particles of CHA0, confirming that the receptor of the tailocin #1 is an O-PS and not a membrane protein nor a part of the core LPS (Fig. 2b, Supplementary Figure 3a). As expected, the deletion of either OBC1 or OBC2 did not influence the sensitivity of Pf-5 (Fig. 2b). Remarkably, when either the OBC4 or OBC5 O-PS were removed (Δ*obc4* or Δ*obc5*), the mutants were more sensitive to the tailocin #1 than the wild-type (Fig. 2b). Conversely, Δ*obc4*Δ*obc5*, lacking both OBC4 and OBC5 O-PS, is completely resistant to the tailocin #1 (Fig. 2b). We also tested extracts from the wild-type CHA0 (containing the two tailocins, the siphovirus and the myovirus), CHA0 T#2 (containing only the tailocin #2) and a CHA0 strain void of phages and tailocins onto these same LPS mutants (Supplementary Figure 3). As expected, we observed the same sensitivity patterns for the full phage(-related) particle extracts (containing tailocins and phages) from the wild-type CHA0 as for the purified tailocin #1 alone (Supplementary Figure 3a). Conversely, the extracts of the tailocin #2 and the strain depleted from tailocins and phages did not exhibit any activity (Supplementary Figure 3b, 3c).

Taken together these results show that Pf-5 harbors four different O-PS types, of which the OBC4 and OBC5 forms are the most widespread on the cell surface of Pf-5 under the tested conditions. The tailocin #1 of CHA0 recognizes both OBC4 and OBC5 O-PS as when they are removed (Δ*obc4*Δ*obc5*), Pf-5 becomes completely resistant.

### The deletion of the major O-PS structures overturns the winning trend of Pf-5 in vitro

Once we identified that the OBC4 and OBC5 O-PS structures have an effect on the resistance of Pf-5 against the tailocin #1 in spot assays, we wanted to see if their deletion would also have an influence on the competitiveness of Pf-5 in direct interbacterial competition.

**Fig. 3:**
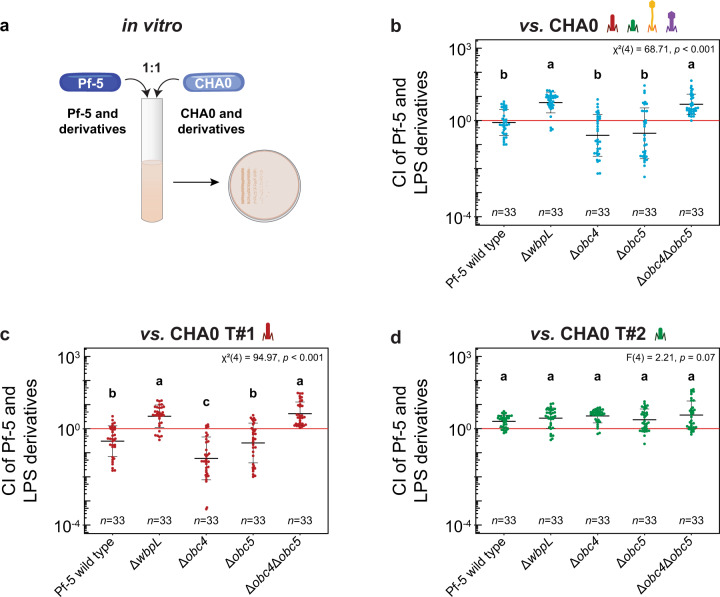
The removal of the OBC4 and OBC5 O-PS clusters makes Pf-5 resistant to the tailocin #1 in competitions with CHA0 in batch culture. The competitive indices (CI) of wild-type Pf-5 and LPS mutant derivatives (Δ*obc4*, Δ*obc5*, Δ*wbpL*, Δ*obc4*Δ*obc*5) were assessed in 1:1 ratio mixtures (**a**) with the CHA0 wild type (**b**) and derivatives Δtail2ΔmyoΔsiph producing exclusively the tailocin #1, CHA0 T#1 (**c**) and Δtail1ΔmyoΔsiph producing exclusively the tailocin #2, CHA0 T#2 (**d**). The red line indicates a competition where both strains would not be influenced by the presence of one another. Statistical differences were assessed by ANOVA and Kruskal-Wallis test using a Bonferroni correction and are indicated with letters a, b and c. Eleven biological independent experiments were performed each with three technical replicates were performed. The horizontal lines indicate the interquartile range with the center representing the median.

We confronted Pf-5 wild-type and LPS mutant derivatives, Δ*wbpL*, Δ*obc4*, Δ*obc5* and Δ*obc4*Δ*obc5* with CHA0 derivatives, specifically a wild-type strain, one producing only the tailocin #1 (Δtail2ΔmyoΔsiph, hereafter “CHA0 T#1”) and one producing only the tailocin #2 (Δtail1ΔmyoΔsiph, hereafter “CHA0 T#2”) in 1:1 ratio competitions in liquid culture (Fig. 3a). The different CI were calculated following CFU counting after 24 h of pairwise competition [10]. Prior to testing the competitiveness of the strains, no growth defects were detected for the different Pf-5 mutants (Supplementary Figure 4), nor for the CHA0 mutant strains [18].

When both wild-type strains were confronted, the results show a 1:1 final ratio meaning that both strains do not outcompete each other (Fig. 3b). Conversely, the two Pf-5 mutants exhibiting uncapped LPS profiles, i.e., Δ*wbpL* and Δ*obc4*Δ*obc5*, significantly outcompeted CHA0, while Δ*obc4* and Δ*obc5* single cluster mutants had a decreased fitness compared to the wild-type Pf-5, although this difference was not significant (Fig. 3b). Against the CHA0 strain only producing the tailocin #1 (Fig. 3c), Pf-5 had a significant decrease in fitness compared to when it was in competition with the wild-type CHA0 (Fig. 3b). Furthermore, as in the competition with the CHA0 wild-type, Δ*wbpL* and the O-PS double mutant Δ*obc4*Δ*obc5* significantly outcompeted CHA0 T#1 (Fig. 3c). Also, there was significantly reduced fitness for the single cluster mutant Δ*obc4* compared to wild-type Pf-5 (Fig. 3c). Finally, there were no differences in the competitions against CHA0 T#2 congruent with the fact that tailocin #2 does not target Pf-5 (Fig. 3d).

These results demonstrate the importance of OBC4 and OBC5 O-PS as receptors for the tailocin #1 of CHA0 during in vitro competitions as when they are both removed, Pf-5 becomes resistant to this tailocin.

### The dual role of the OBC5 O-PS in hemolymph invasion and tailocin resistance

**Fig. 4:**
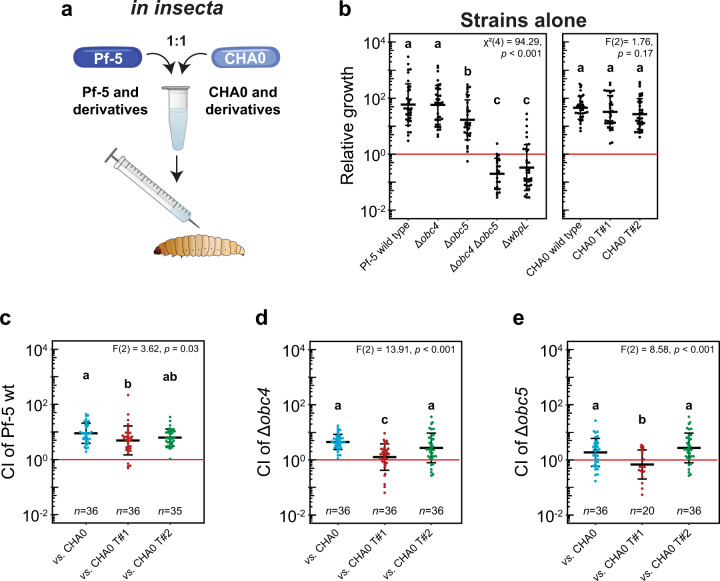
OBC4 and OBC5 contribute to tailocin resistance of *P. protegens* Pf-5 during intraspecific competition in the hemolymph of insects. The relative growth of Pf-5 and CHA0 and their mutant derivatives was assessed in *Galleria mellonella* fourth instar larvae (**a**) following injection of the strains alone (**b**). The competitive index (CI) of Pf-5 (**c**) and LPS mutant derivatives, Δobc4 (**d**), Δobc5 (**e**), were assessed in 1:1 ratio mixtures with CHA0 (blue) and derivatives (Δtail2ΔmyoΔsiph producing exclusively the tailocin #1, CHA0 T#1, red; Δtail1ΔmyoΔsiph producing exclusively the tailocin #2, CHA0 T#2, green) in the larvae. The red line indicates a competition where both strains would not be influenced by the presence of one another. Statistical differences were assessed by ANOVA and Kruskal-Wallis with a Bonferroni correction and are indicated with letters a, b, c and d. Six biological replicates with each six technical replicates were performed, thus, 36 larvae were injected in total. The horizontal lines indicate the interquartile range with the center representing the median.

As *Pseudomonas* strains have been found to not only be able to colonize plants but also insects [13], we assessed the impact of LPS mutation on the fitness of Pf-5 and its ability to compete against a kin strain producing deleterious viral weaponry *in insecta*. Therefore, we injected fourth instar *Galleria mellonella* larvae, with wild-type and mutant strains alone as well as with different pairwise competition mixes in 1:1 ratio between Pf-5 or LPS mutant derivatives (i.e., wild-type, Δ*wbpL*, Δ*obc4*, Δ*obc5* and Δ*obc4*Δ*obc5*) and CHA0 or tailocin mutant derivatives (*i.e*., wild-type, CHA0 T#1 and CHA0 T#2) (Fig. 4a).

While CHA0 and derivatives did not show any significant differences in their ability to colonize the hemolymph of the insect, Pf-5 mutants exhibiting uncapped LPS profiles (Δ*wbpL*, Δ*obc4*Δ*obc5)* showed a drastic loss of fitness when in the insect hemolymph (Fig. 4b). This suggests that the immune system recognizes Pf-5 when it harbors an uncapped LPS profile and dramatically reduces its hemolymph colonization ability, as demonstrated previously for CHA0 where uncapped mutants became sensitive to antimicrobial peptides (AMPs), notably cecropins, that are central to the insect immune defense [11]. Capped wild-type Pf-5 is highly resistant to the AMPs like wild-type CHA0 [11]. Furthermore, the Pf-5 Δ*obc5* mutant also displayed a reduction in fitness when injected alone compared to the wild-type (Fig. 4b), implying that OBC5 O-PS is important to prevent the recognition of Pf-5 by the immune system of the insect. Once in competition with CHA0 and relatives, the growth rates of all Pf-5 strains were significantly reduced (Supplementary figure 5). This could reflect the sharing of niche resources between both strains. Furthermore, the fitness of Pf-5 wild-type and derivatives were significantly reduced when faced with the CHA0 strain that produces only the tailocin #1 compared to the wild-type or the CHA0 that produces only the tailocin #2 (Fig. 4c–e). The impact of the tailocin #1 was even more pronounced when the OBC5 mutant was confronted with CHA0 T#1 as, for the only time in all these *in insecta* competitions the general trend was reversed and a CHA0 derivative was able to outcompete a Pf-5 strain (Fig. 4e). Furthermore, as both Pf-5 and CHA0 are known plant colonizers, we determined the effect of tailocin production by CHA0 targeting Pf-5 during root colonization (Supplementary Figure 6). Conversely to the experiment performed *in insecta*, there was no difference between either the fitness and the growth rates in competition (Supplementary Figures 7c–f and 8) or the growth rates for the strains alone (Supplementary Figure 7b) *in planta*. Thus, in the insect environment, the tailocin #1 of CHA0 and the immune system of the insect host both contribute to a strong decrease in fitness of the LPS mutants of Pf-5.

### Lipopolysaccharides are double-edged swords in tailocin and phage particles recognition

**Fig. 5:**
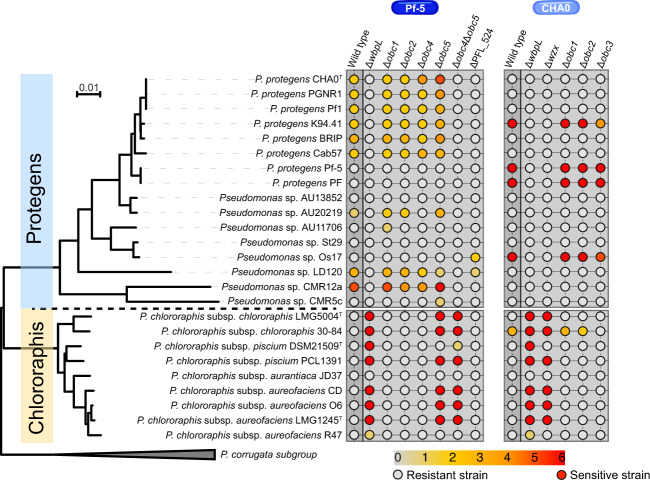
Sensitivity and resistance patterns of different LPS mutant derivatives of CHA0 and Pf-5 towards tailocins and phages extracted from phylogenetically closely related strains belonging to the Pseudomonas protegens and Pseudomonas chlororaphis subgroups. Bacterial lawns from CHA0, Pf-5 and their LPS mutant derivatives were challenged with phage(-related) particles (i.e., tailocins and/or temperate phages) extracted from P. protegens and P. chlororaphis subgroups strains. Sensitivity is demonstrated by the circles colored from grey (resistant) to red (sensitive). The level of sensitivity (0–6) was determined by serially diluting the phage(-related) particle extract at a ratio of 1:4 at each dilution step. A level of sensitivity of six indicates that the strain was sensitive to the extract diluted to 1/4096.

On a larger scale, we wanted to see if the deletions of genes implicated in the production of LPS could influence the sensitivity of CHA0 and Pf-5 towards the phage(-related) particles (i.e., tailocins and temperate phages) of phylogenetically closely related strains. To do so, we exposed different LPS mutants of Pf-5 and CHA0 to the phage(-related) particles extracted from strains belonging to the *P. protegens* (Pp) and *P. chlororaphis* (Pc) subgroups (Fig. 5).

CHA0 and Pf-5 were most sensitive to the tailocins and phages extracted from strains belonging to the same subgroup, *i.e*., to the Pp subgroup, while they were generally resistant to the extracts from Pc subgroup strains (Fig. 5, [18]). Remarkably, this trend completely reversed when genes required for the biosynthesis of a functional LPS core (*wbpL* in CHA0 and *wbpL* and *PFL_0524* in Pf-5) or the transport of the dominant short O-PS (*wzx* in CHA0, [11]) were deleted (Fig. 5). The Δ*wbpL* mutants and Δ*PFL_0524* exhibiting uncapped profiles were protected from the tailocins and phages extracted from strains belonging to their own subgroup, i.e., Pp. However, the Δ*wbpL* mutants were sensitive to the phage(-related) particles extracted from strains belonging to a different subgroup, *i.e*., Pc, while Δ*PFL_0524* remains resistant (Fig. 5).

When focusing on the Pf-5 LPS mutants, we see that Δ*obc1*, Δ*obc2* and Δ*obc4* had largely the same sensitivity profile as the wild-type Pf-5, thus being sensitive to the tailocins and phages from the Pp strains (Fig. 5). By contrast, Δ*obc5* was also sensitive to the tailocins and phages from the Pc strains. This suggests the potential involvement either direct or indirect of the OBC5 in the resistance against these phage(-related) particles. The uncapped double mutant Δ*obc4*Δ*obc5* like Δ*wbpL* was no longer sensitive to the extract of Pp strains while remaining sensitive to the tailocins and phages of the Pc strains (Fig. 5). The Δ*PFL_0524* mutant was resistant to almost all extracts, demonstrating the crucial role of an intact LPS core in the recognition of tailocins and phages (Fig. 5). Therefore, LPS structures appear to play a role for both the protection against phage(-related) particles produced by distant *Pseudomonas* (i.e., Pc) while being receptors for the phage(-related) particles released by closely related strains (*i.e*., Pp).

## Discussion

When confronted with the resident microbiota, environmental *Pseudomonas* strains will rely on a vast arsenal of weapons that vary in the breadth of their spectra, amongst them narrow-spectrum phage-related weaponry. Here, we performed a detailed study on the receptors for specific kin-targeting R-tailocins in phylogenetically very closely related strains and on how receptor-tailocin interactions affect the fitness of these strains in the host environment. We focused on two widely used model environmental pseudomonads, CHA0 and Pf-5, that share 98.9% of their genomes (coverage 92.9%), but still differ in their production of phage(-related) particles (tailocins and phages) and their sensitivity profile towards the phage(-related) particles of each other and other kin strains (Fig. 1, [18]). We identified through a Tn-seq approach that among the genes that differ between both strains, genes and clusters involved in the LPS profile of Pf-5 account for the sensitivity of this strain towards one of the R-tailocins (tailocin #1) of its kin, CHA0 (Fig. 1). This result is in line with the identification of LPS as receptors for tailocins in other *Pseudomonas* species [25, 35, 44, 45] or even a different genus [46]. Indeed, as the outer membrane of Gram-negative bacteria is essentially composed of LPS [36], it is not surprising that these cell-surface decorations act as receptors for phage tail-like particles in pseudomonads, like they do for bacteriophages [47].

To have a better understanding of the targets for the tailocin #1 of CHA0, we investigated the LPS profile of Pf-5. Through genetic analysis, we identified four different O-PS clusters, OBC1, OBC2, OBC4 and OBC5, in addition to the clusters and genes involved in the formation of the core oligosaccharide-lipid A structure (Fig. 1). Deletion mutants of OBC1 and OBC2 did not have a different LPS or sensitivity profile compared to the wild-type Pf-5 (Fig. 2a), a phenotype that we previously observed also in CHA0 for these two O-PS types [11]. It is possible that these clusters may not encode specific O-PS structures but have a role in modifying these structures depending on the environment and the different stress factors that the bacterium is exposed to [6, 11]. Conversely, deletion mutants of the OBC4 and OBC5 had downshifts in their banding patterns compared to the wild-type Pf-5 (Fig. 2a). We conclude that the OBC4 encodes a long O-PS while the OBC5 encodes a mid-length O-PS. Interestingly, the Δ*obc5* mutant also had lost the band above the core-oligosaccharide-lipid A. This phenotype resembles that of the Δ*wbpL* and Δ*PFL_0524* mutants, leading us to hypothesize that genes within the OBC5 have an effect on the core, complementing those present in the conserved core-oligosaccharide-lipid A gene clusters.

Furthermore, when individual genes were mutated within the OBC4 or OBC5 cluster, this had an effect on the other O-PS structure (Supplementary Figure 2). This suggests that both OBC4 and OBC5 O-PS share proteins for their synthesis and/or export, indicating a possible crosstalk between both gene clusters that could permit a higher plasticity in the LPS profile. Indeed, the layer formed by LPS structures around Gram-negative bacteria is important for protection for example against antibiotics or AMPs [11]. However, bacteria need this LPS barrier to be malleable to interact with their environment more efficiently. Therefore, bacteria have evolved mechanisms and enzymes to customize their LPS profiles to adapt to the colonized environment [48]. Furthermore, within a population, it is common to find bacteria with multiple different phenotypic LPS profiles [49]. Therefore, it is possible that Pf-5 within different environments could exhibit different LPS profiles. Accordingly, phages and tailocins that specifically target Pf-5 could have adapted to target both OBC4 and OBC5 O-PS structures, explaining why Δ*obc4* and Δ*obc5* are both sensitive to the tailocin #1 of CHA0.

In addition to being involved in the interaction with phages, LPS are also important for the interaction with a potential bacterial host. Bacterial human pathogens such as *Pseudomonas aeruginosa*, *Helicobacter pylori* or *Salmonella enterica* have been found to modify their LPS structures to increase virulence to better colonize their host, modulate the immune system or even avoid recognition by the immune system of their host [48, 50]. In insects, bacteria use similar mechanisms to colonize their host [11]. Furthermore, it was found that *Pseudomonas* mutants exhibiting uncapped LPS profiles had a decreased insecticidal activity and insect invasion capacity compared to the wild-types owing to their drastically higher sensitivity to insect AMPs, demonstrating the importance of LPS structures in the virulence of these strains [11]. Here, we found that strains that lack O-PS structures have a decreased colonization ability of the insect hemolymph likely because they are more easily detected by the immune system (Fig. 4). Furthermore, we found that there is a significant decrease of the growth rates of the Δ*obc4* and Δ*obc5* mutants in all competitions *in insecta* compared to when they colonized the insect alone (Supplementary Figure 5). The fitness was even more reduced when the mutants were in competition with CHA0 T#1 that produces only the tailocin #1. For Δ*obc5* this trend was drastic enough that it was no longer able to win against CHA0 T#1. These observations can be explained by the fact that the tailocins of CHA0 (specifically the tailocin #1) and the immune system of the insect host act in tandem both contributing to a decreased fitness of the LPS mutants of Pf-5. Thus, the bacterium is confronted with a trade-off, as without LPS structures it is resistant to the tailocins of a kin competitor but unable to evade the immune system of the insect, whereas with LPS structures the bacterium can hide from the immune system of the insect but is sensitive to the phage tail-like particles of its kin.

**Fig. 6:**
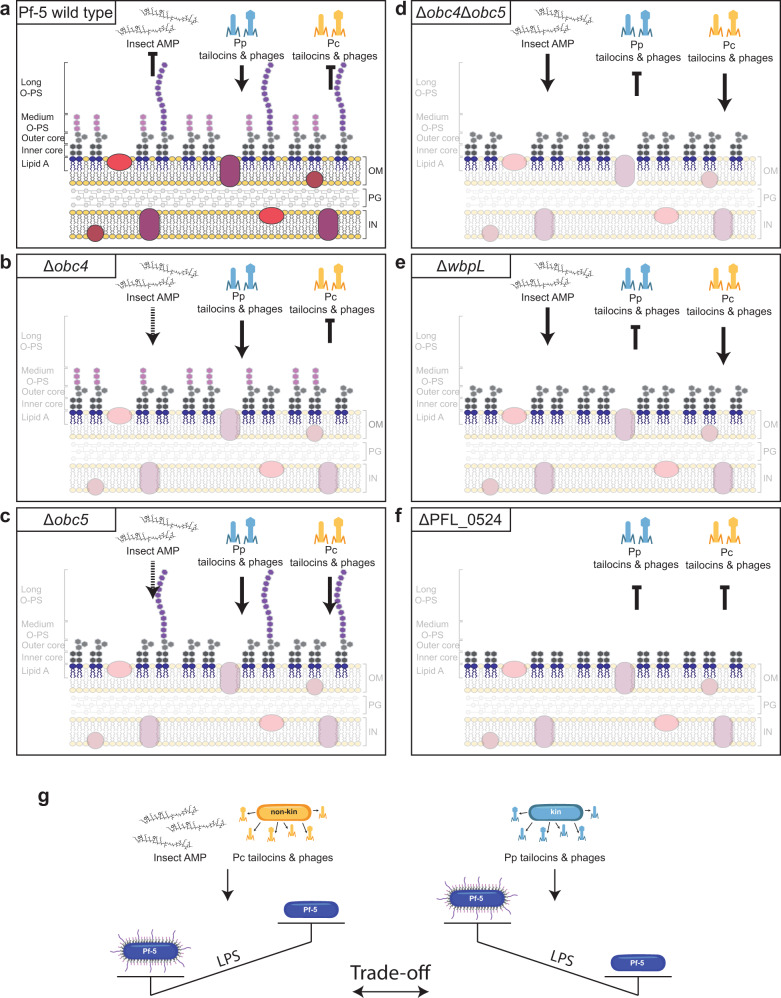
The trade-off between being virulent in insecta and resistant to the phage(-related) particles of non-kin strains, or being resistant to the phage(-related) particles of kin strains. The cartoons explain the results obtained following the various competition assays from this study on the different Pf-5 LPS mutant derivatives (**a**, Pf-5 wild-type; **b**, Δobc4; **c**, Δobc5; **d**, Δobc4Δobc5; **e**, ΔwbpL; **f**, ΔPFL_0524). **g** Model demonstrating the trade-off that Pf-5 faces when confronted with different environments and their inhabitants. Lipid A: dark blue, inner core: dark grey, outer core: light grey, O-PS: purple (OBC4) and pink (OBC5). To represent the immune defense of the insect, the structure of cecropin A, an antimicrobial peptide (AMP, [11]), is shown. Phylogenetically closely related bacteria and phage(-related) particles produced by these kin strains are shown in light blue. Phylogenetically distantly related bacteria and phage(-related) particles produced by these non-kin strains are shown in yellow/orange.

LPS structures are also important for the interaction with phylogenetically more distantly related *Pseudomonas* strains. Indeed, Pf-5 and CHA0 wild-type strains were generally sensitive to the tailocins and temperate phages extracted from Pp strains, while they were resistant to those extracted from Pc strains (Fig. 5, Fig. 6a, [18]). However, this trend was inverted when they exhibited uncapped LPS profiles (Δ*wbpL*, Fig. 5, Fig. 6e, and Δ*obc4*Δ*obc5*, Fig. 5, Fig. 6d). When we specifically focused on the Pf-5 mutants, Δ*obc4* had the same sensitivity profile as the wild-type (Fig. 5, Fig. 6b). Conversely, for Δ*obc5* we found that it is sensitive to almost all phage(-related) particles (Fig. 5, Fig. 6c). Finally, Δ*PFL_0524* was resistant to almost all phage(-related) particles (Fig. 5, Fig. 6f). Thus, we suggest that Pp tailocins and phages specifically interact with the OBC4 and/or OBC5 O-PS structures (Fig. 6). Pc tailocins and phages would interact with the core as when it was exposed (Δ*wbpL*, Δ*obc4*Δ*obc5*), Pf-5 was sensitive while when there was a reduced core (Δ*PFL_0524*), Pf-5 became resistant (Fig. 6). We speculate that the OBC5 O-PS type is more abundant than the OBC4 type as Δ*obc4* was more resistant than Δ*obc5*. Thus, OBC5 O-PS would hide and protect the core structure lacking OBC4 from the Pc tailocins and phages. Therefore, LPS are double-edge swords that either permit the protection from the phage(-related) particles from distantly related strains or are recognized by the phage(-related) particles from closely related strains. However, we previously found considerable diversity in clusters encoding tailocins and temperate phages among the tested strains [18], which may explain the differential sensitivities of CHA0, Pf-5 wild types and mutants with respect to their LPS equipment.

Our findings support the idea that LPS may function as both receptors and defense structures against tailocins and phages released by phylogenetically closely related bacterial strains. Indeed, we showed that two genetically highly similar strains have different sensitivity patterns towards the same phage(-related) particle. Furthermore, we showed that a single phage tail-like particle can have multiple receptors. This could be an evolutionary response where bacteria can interchange their cell surface structures according to the environment. Finally, we showed that Pf-5 faces a trade-off between being resistant to the tailocins of a kin and evading the immune system of the insect (Fig. 6g). These findings show the intricate relationship between different bacterial strains, their potential hosts and potential competitors.

## Supplementary information

Supplementary material

## Data Availability

The generated Tn-seq Datasets were deposited on EBI platform, sample accession number: ERS5375177-ERS5375182 under the BioProject PRJEB41502. The raw data of this manuscript are available on the Dryad platform. Heiman, Clara et al. (2022), Pivotal role of O-antigenic polysaccharide display in the sensitivity against phage tail-like particles in environmental Pseudomonas kin competition., Dryad, Dataset, 10.5061/dryad.1zcrjdft6

## References

[1] Keel C (2016). A look into the toolbox of multi-talents: insect pathogenicity determinants of plant-beneficial pseudomonads. Environ Microbiol.

[2] Kupferschmied P, Maurhofer M, Keel C (2013). Promise for plant pest control: root-associated pseudomonads with insecticidal activities. Front Plant Sci.

[3] Vesga P, Augustiny E, Keel C, Maurhofer M, Vacheron J (2021). Phylogenetically closely related pseudomonads isolated from arthropods exhibit differential insect-killing abilities and genetic variations in insecticidal factors. Environ Microbiol.

[4] Flury P, et al. Persistence of root-colonizing *Pseudomonas protegens* in herbivorous insects throughout different developmental stages and dispersal to new host plants. ISME J. 2018;14:860–72. 10.1038/s41396-018-0317-4 PMC6461868 30504899

[5] Flury P (2016). Insect pathogenicity in plant-beneficial pseudomonads: phylogenetic distribution and comparative genomics. ISME J.

[6] Vesga P (2020). Transcriptome plasticity underlying plant root colonization and insect invasion by *Pseudomonas protegens*. ISME J.

[7] Ramette A (2011). *Pseudomonas protegens* sp. nov., widespread plant-protecting bacteria producing the biocontrol compounds 2,4-diacetylphloroglucinol and pyoluteorin. Syst Appl Microbiol.

[8] Smits THM, et al. Updated genome sequence and annotation for the full genome of *Pseudomonas protegens* CHA0. Microbiol Resour Announc. 2019;8:e01002-19. 10.1128/MRA.01002-19 PMC6763652 31558637

[9] Flury P (2019). Persistence of root-colonizing *Pseudomonas protegens* in herbivorous insects throughout different developmental stages and dispersal to new host plants. ISME J.

[10] Vacheron J (2019). T6SS contributes to gut microbiome invasion and killing of an herbivorous pest insect by plant-beneficial *Pseudomonas protegens*. ISME J.

[11] Kupferschmied P (2016). Specific surface glycan decorations enable antimicrobial peptide resistance in plant-beneficial pseudomonads with insect-pathogenic properties. Environ Microbiol.

[12] Kupferschmied P, Péchy-Tarr M, Imperiali N, Maurhofer M, Keel C (2014). Domain shuffling in a sensor protein contributed to the evolution of insect pathogenicity in plant-beneficial Pseudomonas protegens. PLOS Pathog.

[13] Péchy-Tarr M (2013). Control and host-dependent activation of insect toxin expression in a root-associated biocontrol pseudomonad. Environ Microbiol.

[14] Ruffner B (2013). Oral insecticidal activity of plant-associated pseudomonads. Environ Microbiol.

[15] Péchy-Tarr M (2008). Molecular analysis of a novel gene cluster encoding an insect toxin in plant-associated strains of *Pseudomonas fluorescens*. Environ Microbiol.

[16] Yan Q, et al. Secondary metabolism and interspecific competition affect accumulation of apontaneous mutants in the GacS-GacA regulatory system in *Pseudomonas protegens*. mBio. 2018;9:e01845-17. 10.1128/mBio.01845-17 PMC5770548 29339425

[17] Keel C (1992). Suppression of root diseases by *Pseudomonas fluorescens* CHA0: importance of the bacterial secondary metabolite 2,4-diacetylphloroglucinol. Mol Plant-Microbe Interact.

[18] Vacheron J, Heiman CM, Keel C Live cell dynamics of production, explosive release and killing activity of phage tail-like weapons for *Pseudomonas* kin exclusion. Commun Biol. 2021;4:87. 10.1038/s42003-020-01581-1 PMC7815802 33469108

[19] Ghequire MGK, De Mot R (2014). Ribosomally encoded antibacterial proteins and peptides from *Pseudomonas*. FEMS Microbiol Rev.

[20] Bruce JB, West SA, Griffin AS (2017). Bacteriocins and the assembly of natural *Pseudomonas fluorescens* populations. J Evol Biol.

[21] Michel-Briand Y, Baysse C (2002). The pyocins of *Pseudomonas aeruginosa*. Biochimie.

[22] Ghequire MGK, De, Mot R (2015). The tailocin tale: peeling off phage tails. Trends Microbiol.

[23] Scholl D (2017). Phage tail–like bacteriocins. Annu Rev Virol.

[24] Dorosky RJ, Pierson LS, Pierson EA*Pseudomonas chlororaphis* produces multiple R-tailocin particles that broaden the killing spectrum and contribute to persistence in rhizosphere communities. Appl Environ Microbiol. 2018;84:e01230–18. 10.1128/AEM.01230-18 PMC6121977 30030224

[25] Carim S, et al. Systematic discovery of pseudomonad genetic factors involved in sensitivity to tailocins. ISME J. 2021;15:2289-305. 10.1038/s41396-021-00921-1 PMC8319346 33649553

[26] Hockett KL, Renner T, Baltrus DA (2015). Independent co-option of a tailed bacteriophage into a killing complex in. Pseudomonas mBio.

[27] Nakayama K (2000). The R-type pyocin of *Pseudomonas aeruginosa* is related to P2 phage, and the F-type is related to lambda phage. Mol Microbiol.

[28] Ghequire MGK (2015). Different ancestries of R-tailocins in rhizospheric *Pseudomonas* isolates. Genome Biol Evol.

[29] Patz S, et al. Phage tail-like particles are versatile bacterial nanomachines – A mini-review. J Adv Res. 2019. Elsevier B.V., 19: 75–84 10.1016/j.jare.2019.04.003 PMC6629978 31341672

[30] Desfosses A (2019). Atomic structures of an entire contractile injection system in both the extended and contracted states. Nat Microbiol.

[31] Ge P (2015). Atomic structures of a bactericidal contractile nanotube in its pre- and postcontraction states. Nat Struct Mol Biol.

[32] Nobrega FL (2018). Targeting mechanisms of tailed bacteriophages. Nat Rev Microbiol.

[33] Fernandez M, Godino A, Príncipe A, Morales GM, Fischer S (2017). Effect of a *Pseudomonas fluorescens* tailocin against phytopathogenic *Xanthomonas* observed by atomic force microscopy. J Biotechnol.

[34] Fraser A (2021). Quantitative description of a contractile macromolecular machine. Sci Adv.

[35] Köhler T, Donner V, van Delden C (2010). Lipopolysaccharide as shield and receptor for R-pyocin-mediated killing in *Pseudomonas aeruginosa*. J Bacteriol.

[36] Huszczynski SM, Lam JS, Khursigara CM. The role of *Pseudomonas aeruginosa* lipopolysaccharide in bacterial pathogenesis and physiology. Pathogens. 2020;1:6. 10.3390/pathogens9010006 PMC7168646 31861540

[37] Lam JS, Taylor VL, Islam ST, Hao Y, Kocíncová D. Genetic and functional diversity of *Pseudomonas aeruginosa* lipopolysaccharide. Front Microbiol. 2011;2:118. 10.3389/fmicb.2011.00118 PMC3108286 21687428

[38] Larsen RA, Wilson MM, Guss AM, Metcalf WW (2002). Genetic analysis of pigment biosynthesis in *Xanthobacter autotrophicus* Py2 using a new, highly efficient transposon mutagenesis system that is functional in a wide variety of bacteria. Arch Microbiol.

[39] Edgar RC (2004). MUSCLE: multiple sequence alignment with high accuracy and high throughput. Nucleic Acids Res.

[40] Guindon S (2010). New algorithms and methods to estimate maximum-likelihood phylogenies: assessing the performance of PhyML 3.0. Syst Biol.

[41] Martínez-García E, de Lorenzo V (2011). Engineering multiple genomic deletions in Gram-negative bacteria: analysis of the multi-resistant antibiotic profile of *Pseudomonas putida* KT2440. Environ Microbiol.

[42] Davis MRJ, Goldberg JB. Purification and visualization of lipopolysaccharide from Gram-negative bacteria by hot aqueous-phenol extraction. JoVE (Journal Vis Exp). 2012;e3916. 10.3791/3916. 10.3791/3916 PMC3466933 22688346

[43] Woodward R (2010). In vitro bacterial polysaccharide biosynthesis: defining the functions of Wzy and Wzz. Nat Chem Biol.

[44] Buth SA, Shneider MM, Scholl D, Leiman PG. Structure and analysis of R1 and R2 pyocin receptor-binding fibers. Viruses. 2018;8:427. 10.3390/v10080427 PMC6116203 30110933

[45] Bertozzi Silva J, Storms Z, Sauvageau D (2016). Host receptors for bacteriophage adsorption. FEMS Microbiol Lett.

[46] Yao GW, et al. A broadhost-range tailocin from Burkholderia cenocepacia. Appl Environ Microbiol. 2017;83:e03414-16. 10.1128/AEM.03414-16 PMC5411513 28258146

[47] De Smet J, Hendrix H, Blasdel BG, Danis-Wlodarczyk K, Lavigne R. Pseudomonas predators: understanding and exploiting phage–host interactions. Nat Rev Microbiol. 2017;15:517–530. 10.1038/nrmicro.2017.61 28649138

[48] Simpson BW, Trent MS (2019). Pushing the envelope: LPS modifications and their consequences. Nat Rev Microbiol.

[49] Lerouge I, Vanderleyden J (2002). O-antigen structural variation: mechanisms and possible roles in animal/plant-microbe interactions. FEMS Microbiol Rev.

[50] Maldonado RF, Sá-Correia I, Valvano MA (2016). Lipopolysaccharide modification in Gram-negative bacteria during chronic infection. FEMS Microbiol Rev.

