# Supplementary material for: Pivotal role of O-antigenic polysaccharide display in the sensitivity against phage tail-like particles in environmental Pseudomonas kin competition
Source: ISME J. 2022 Mar 10;16(7):1683–93. doi: 10.1038/s41396-022-01217-8 (PMC9213528; doi:10.1038/s41396-022-01217-8)
Supplement: Supplementary file 1 — Supplementary material [file 41396_2022_1217_MOESM1_ESM.docx]

**SUPPLEMENTARY INFORMATION**

**The pivotal role of O-antigenic polysaccharide display in the sensitivity against phage tail-like particles in environmental Pseudomonas kin competition**

**Clara Margot Heiman^1^, Monika Maurhofer^2^, Sandra Calderon^3^, Mélanie Dupasquier^3^, Julien Marquis^3^, Christoph Keel^1#^** and **Jordan Vacheron^1#^**

^1^ Department of Fundamental Microbiology, University of Lausanne, Lausanne, Switzerland; ^2^ Plant Pathology, Institute of Integrative Biology, Swiss Federal Institute of Technology (ETH) Zurich, Zurich, Switzerland; ^3^ Lausanne Genomic Technologies Facility, University of Lausanne, Lausanne, Switzerland

Corresponding authors: Christoph Keel (email: christoph.keel@unil.ch) and Jordan Vacheron (email: jordan.vacheron@unil.ch), Department of Fundamental Microbiology, University of Lausanne, Biophore Building, CH-1015 Lausanne, Switzerland.

**Supplementary Methods**

**Tn-seq DNA library preparation and Illumina sequencing**

**Sequence processing and statistical analysis**

**Bacterial competition assay *in vitro*, *in insect* and *in planta***

**Supplementary Results**

**The absence of the OBC4 and OBC5 O-PS structures decreases the competitiveness of Pf-5 against CHA0 *in planta*.**

**Supplementary Figures**

**Supplementary Figure 1.** Gene clusters involved in LPS formation in CHA0 and Pf-5.

**Supplementary Figure 2.** LPS profile of Pf-5 and mutants with deletions in the OBC4 and OBC5 gene clusters.

**Supplementary Figure 3.** Pf-5 and its LPS mutant derivatives are resistant to the tailocin #2 of CHA0.

**Supplementary Figure 4.** Growth kinetics of Pf-5 and LPS derivatives.

**Supplementary Figure 5.** The competition with *P. protegens* CHA0 and derivatives reduces growth rates of *P. protegens* Pf-5 and derivatives *in insecta*.

**Supplementary Figure 6.** Scheme of the plant inoculation experiment.

**Supplementary Figure 7.** The deletion of either OBC4 or/and OBC5 does not affect the competitiveness of *P. protegens* Pf-5 *in planta*.

**Supplementary Figure 8.** The competition with *P. protegens* CHA0 and derivatives has no impact on growth rates of *P. protegens* Pf-5 and derivatives during plant colonization.

**Supplementary Tables**

**Supplementary Table 1.** Bacterial strains used in this study.

**Supplementary Table 2**. CHA0 derivatives, Pf-5 derivatives and *Escherichia coli* strains used in this study.

**Supplementary Table 3**. Plasmids used in this study.

**Supplementary Table 4.** Oligonucleotides used for the construction of the Pf-5 LPS mutant derivatives.

**Supplementary Table 5**. Transposon sequencing characteristics.

**Supplementary datasets**

**Dataset 1.** Differing genes between CHA0 and Pf-5

**Dataset 2.** Tn-Seq Pf-5 *vs.* tailocin #1

**References**

**Supplementary Methods**

**Tn-seq DNA library preparation and Illumina sequencing**

The genomic DNA was processed according to the first step of the TruSeq nano protocol (Illumina). Briefly, 100 ng of genomic DNA was sheared with a Covaris S220 device using the 350-bp insert setting parameters. Following the ligation of TruSeq adapters, the ligation products were purified twice with SPRI beads at a 1X ratio. Half of the purified product was amplified by PCR with a specific primer containing the Illumina P5 sequence (AATGATACGGCGACCACCGAGATCTAC) and a second primer which is specific for the Tn5 transposon sequence, and tailed with an Illumina P7 sequence (CAAGCAGAAGACGGCATACGAGATTTGTGGACAACAAGCCAGGGATGTAACG, the specific sequence of the transposon is underlined). The PCR was performed with the KAPA HiFi HotStart ReadyMix kit (Roche). Cycling conditions were 95 °C for 3 min, followed by 22 cycles of 98 °C for 20 s, 65 °C for 15 s, and 72 °C for 30 s, and a final extension of 5 min at 72 °C. The final library was purified with SPRI beads at a 0.9X ratio and quantified by Qubit (Thermo Scientifics). The size pattern of the final library was analyzed with a fragment analyzer (Agilent).

Paired end sequencing was performed on a HiSeq2500 system (Illumina) in rapid mode with a 14 pM library pool (equimolar for each library) and spiked with 2% PhiX. The read1 was performed for 26 cycles for proper sequencing quality filtering using the ‘read1’ primer containing a standard TruSeq read1 sequence (ACACTCTTTCCCTACACGACGCTCTTCCGATCT, 500 nM in HT1 buffer). Then, the transposon sequencing was performed on read2 for 151 cycles using the ‘read2’ primer containing the standard TruSeq sequence (GTGACTGGAGTTCAGACGTGTGCTCTTCCGATCT) mixed with the custom transposon sequencing primer named Tn5custom (GGTACCGAGCTCGAATTCATCG), both at 500 nM in HT1 buffer. Index sequencing primers tubes were kept untouched. Sequencing data were demultiplexed using the bcl2fastq conversion software (version 2.20, Illumina) and further processed for transposon insertion analysis.

**Sequence processing and statistical analysis**

The raw reads were pre-processed by discarding R1 and trimming the 5’ Tn5 IR sequence (5’ GGTTGAGATGTGTATAAGAGACA 3’) of R2 reads using cutadapt (v.2.3, [1]). The Tn5-IR-free reads were further trimmed for Illumina adapters and low-quality bases using cutadapt, and low complexity tail with reaper (v.15-065, [2]). Following these pre-processing steps, the cleaned-reads were mapped to the genome of *P. protegens* Pf-5 (CP000076.1) using bwa (v.0.7.17, options: -T 0 -a -M, [3]). The number of reads per insertion site was computed using a custom script. Wiggle and bed files were generated. Statistical analysis was performed with TRANSIT (v.2.5.2, [4]). First, insert site counts were normalized using the TTR method [4]. The comparative analysis for determining conditional essentiality of genes was performed using the resampling method (10’000 permutations, options: -s 10000 -n TTR -a [4]). All the figures were drawn using RStudio (v1.1.453). The characteristics of the Tn-seq can be found in Supplementary Table 5.

**Bacterial competition assay *in vitro***

To test the *in vitro* competitiveness of Pf-5 derivatives lacking specific receptors for CHA0 tailocin, we tested wild-type Pf-5 and mutants of interest (*i.e.*, Pf-5 Δ*wbpL*, Pf-5 Δ*obc1*, Pf-5 Δ*obc2*, Pf-5 Δ*obc4*, Pf-5 Δ*obc5* or Pf-5 Δ*obc4*Δ*obc5*, Supplementary Table 2) in pair-wise competition against either a wild-type CHA0, a CHA0 mutant only producing the tailocin #1 (*i.e.*, CHA0 Δtail2ΔmyoΔsiph) and a CHA0 mutant only producing the tailocin #2 (*i.e.*, CHA0 Δtail1ΔmyoΔsiph) in liquid medium following a protocol adapted from [5]. Briefly, exponential growth phase cultures were adjusted to an OD_600_ of 0.1 in fresh NYB medium. Adjusted cultures were placed in 1:1 ratio competition in glass tubes and incubated at 25°C without shaking. Through colony forming units (CFU) counting, the survival of each strain was assessed at t=24 h. To differentiate between the two competing strains, CHA0 and mutant derivatives were labeled with a constitutive *gfp2* tag (Supplementary Table 2), which permitted to also use gentamycin as a selective factor for these strains. The survival of Pf-5 and derivatives was determined by counting of non-*gfp2* strains on NA plates. These data were used to calculate the competitive indices (CI) of Pf-5 and its LPS mutant derivatives following the equation CI=[CFU_Pf-5_24h_/CFU_CHA0_24h_]/ [CFU_Pf-5_0h_/CFU_CHA0_0h_] [6]. Eleven biological replicates with three technical replicates each were performed.

**Bacterial competition assay *in insecta***

To assess the competitiveness of Pf-5 derivatives lacking specific CHA0 tailocin receptors in an insect host, we tested the same strains as detailed above for the *in vitro* assay in injection assays using larvae of *Galleria mellonella*. Cell suspensions for inoculation of the strains of interest were prepared from exponential cultures and adjusted to an OD_600_ of 0.1 and placed in 1:1 ratio competitions immediately prior to injection. Five μL of this competition mix were injected into the third proleg of fourth instar *G. mellonella* larvae. As a control, we injected sterile 0.9% NaCl solution. After 24 h, the larvae were dissected in order to collect the hemolymph containing the bacteria. Briefly, larvae were frozen at -80°C for 15 min. Then, two incisions were made along the pseudopodia to collect the hemolymph. CFU counting was performed in order to calculate the CI for Pf-5 and its LPS mutant derivatives. As above, to differentiate between the two competing strains, CHA0 and mutant derivatives were labeled with a constitutive *gfp2* tag. The survival of Pf-5 and its derivatives at t=24 h was determined by counting the CFU numbers of non-*gfp-2* strains on NA plates. To assess the relative growth of the strains when alone or in competitions, the CFU at t=24 h was divided by the CFU at t=0. Four biological replicates with six technical replicates each were performed.

**Bacterial competition assay *in planta***

To assess the competitiveness of Pf-5 and LPS derivatives on a plant host, we tested the same strains as detailed above for the *in vitro* and *in insecta* assays in microcosms involving wheat seedlings (see scheme in Supplementary Figure 5). Seeds of the wheat cultivar Arina were surface-disinfested using a 4% [v/v] bleach solution for 20 min with shaking at 55 rpm, then thoroughly washed with sterile water and germinated on 1% soft agar (SERVA) plates at room temperature for 24 h (adapted from [7]. Individual seedlings were placed in sterile glass tubes (length 15.5 cm, diameter 1.7 cm) on a layer of 1% SERVA soft agar (6 mL per tube). The bottom of the tubes was covered with aluminum foil to protect the roots growing into the agar from light. For the bacterial inocula, overnight cultures were restarted at 1:50 [v/v] in fresh NYB and grown to an OD at 600 nm of 0.4-0.6. Cells were harvested, suspended in sterile water and adjusted to an OD at 600 nm of 0.1. Aliquots of 50 µl of the cell suspensions of the strains of interest were inoculated either alone or in 1:1 ratio competitions onto the seedlings. Following a six-day incubation period in a growth chamber set to 60% relative humidity and 25°C with a 16 h light period (160 µE/m^2^/s), followed by an 8 h dark period at 18°C, the plants were harvested. The roots were separated from the shoots and the fresh weights measured. The shoots were then placed at 55°C over-night to assess their dry weight. The roots of each tube were crushed in 1 mL of sterile 0.8% NaCl solution using a bead better and the resulting mixture was plated to perform CFU counting in order to calculate the CI for Pf-5 and its LPS mutant derivatives. As above, to differentiate between the two competing strains, CHA0 and mutant derivatives were labeled with a constitutive *gfp2* tag. The survival of Pf-5 and its mutant derivatives at t=6 days was determined by counting the number of CFU of non-*gfp2* strains on NA plates. To assess the relative growth of the strains when alone or in competition, the CFU at t=6 days was divided by the CFU at t=0. Four biological replicates with three technical replicates each were performed.

**Supplementary Results**

**The absence of the OBC4 and OBC5 O-PS structures decreases the competitiveness of Pf-5 against CHA0 *in planta*.** As both Pf-5 and CHA0 are known root colonizers, we were interested to determine if the effect of tailocin production by CHA0 targeting Pf-5 can be assessed during plant colonization. Therefore, we inoculated seedlings of the wheat cultivar Arina with both bacteria in a 1:1 ratio. We used the same CHA0 derivatives as for the liquid competitions (*i.e.*, CHA0, CHA0 T#1 and CHA0 T#2) and used Pf-5 wild-type, Δ*obc4*, Δ*obc5* and Δ*obc4*Δ*obc5* as the Pf-5 derivatives. After six days of incubation, bacteria were collected from the roots to perform CFU counting (Supplementary Figure 5, Supplementary Figure 6a).

Conversely to the experiment performed *in insecta*, we observed no difference between the colonization by the strains alone *in planta* (Supplementary Figure 6b). However, there was an overall trend of decreased fitness of the Pf-5 derivatives in competition with CHA0 derivatives compared to the strains alone (Supplementary Figure 6c-6f). This reduction of fitness could be due to the tailocin #1 of CHA0 or simply be a result of the obligation of sharing the resources. Specifically, in competition against the Pf-5 wild-type, a trend of decreased fitness was visible where the Pf-5 wild type in competition against CHA0 T#1 had a lower fitness compared to the competition with the CHA0 wild type (Supplementary Figure 6c). A similar trend appeared in the competitions with Pf-5 Δ*obc5* (Supplementary Figure 6e). Conversely, the fitness of Pf-5 Δ*obc4* was lower than in competition with wild-type CHA0 compared to CHA0 T#1 (Supplementary Figure 6d). Finally, Pf-5 Δ*obc4*Δ*obc5* always had a decreased fitness in competition *in planta*. The impact of the tailocin appears to be less important in this environment compared to the environment of the insect. This could be the effect of the size of the niche (broader spatialization of the bacteria) and/or the access to and the quantity of nutrients that are more available inside the hemolymph of the insect and scarcer on plant roots.

Overall, all strains, wild-types and mutants, are able to colonize the seedlings in a similar way and the effect of the tailocins on the competition appears to be less influential in the root environment.

**Supplementary Figures**


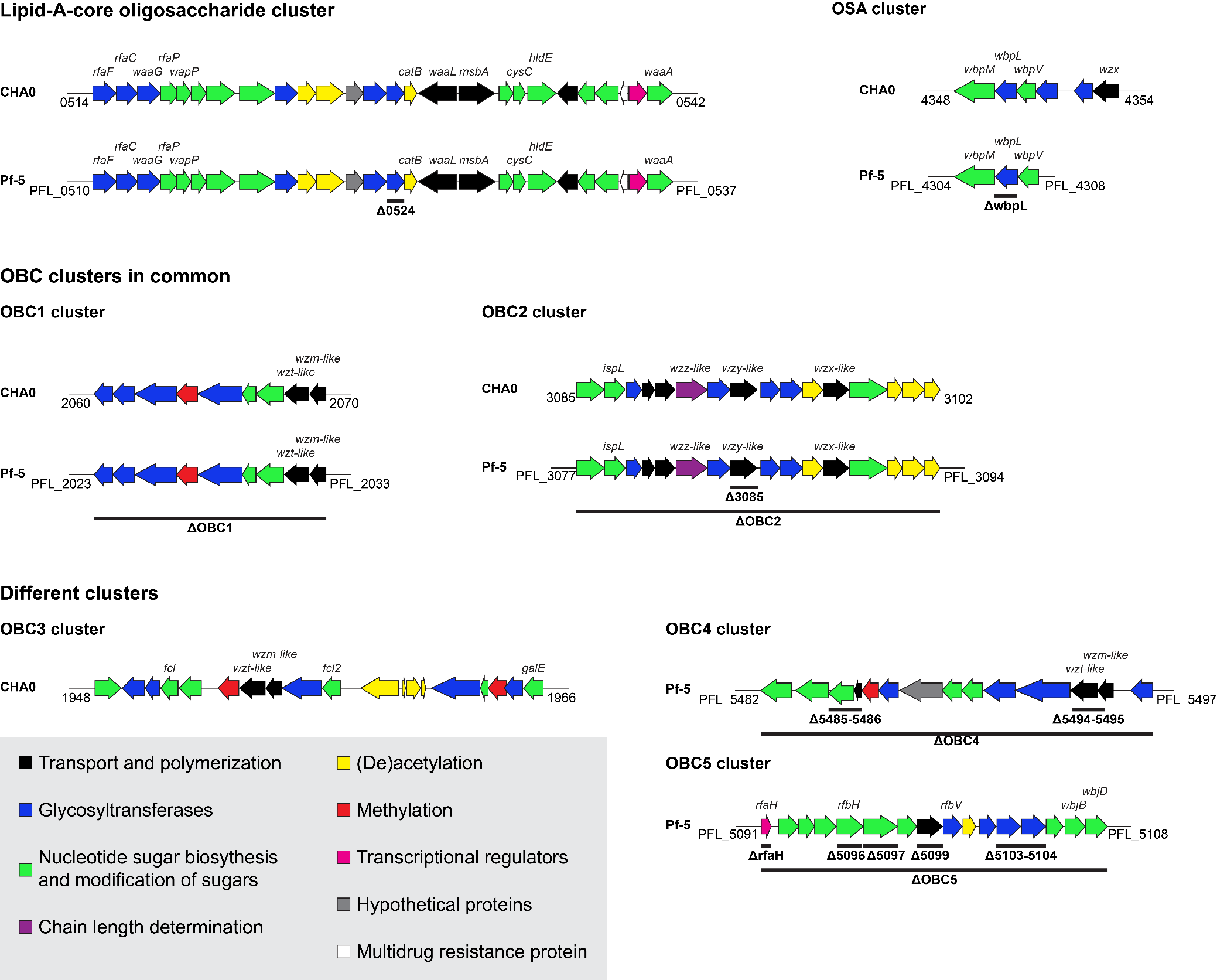


**Supplementary Figure 1. Gene clusters involved in LPS formation in P. protegens strains CHA0 and Pf-5.** From top to bottom and left to right are represented the gene clusters for the lipid-A-core oligosaccharide, the O-specific antigen (OSA) gene cluster, and the different O-antigenic polysaccharide biosynthesis clusters (OBC) present in CHA0 and Pf-5. The OSA gene cluster of CHA0 is reduced compared to the prototype OSA cluster of Pseudomonas aeruginosa, and is further reduced in Pf-5 [8]. The OBC1 and OBC2 gene clusters are present in both CHA0 and Pf-5. The OBC3 gene cluster is only present in CHA0 while the OBC4 and OBC5 gene clusters are only present in Pf-5. The different deletion mutations constructed in Pf-5 are indicated below the clusters. Genes are colored according to their different functions.


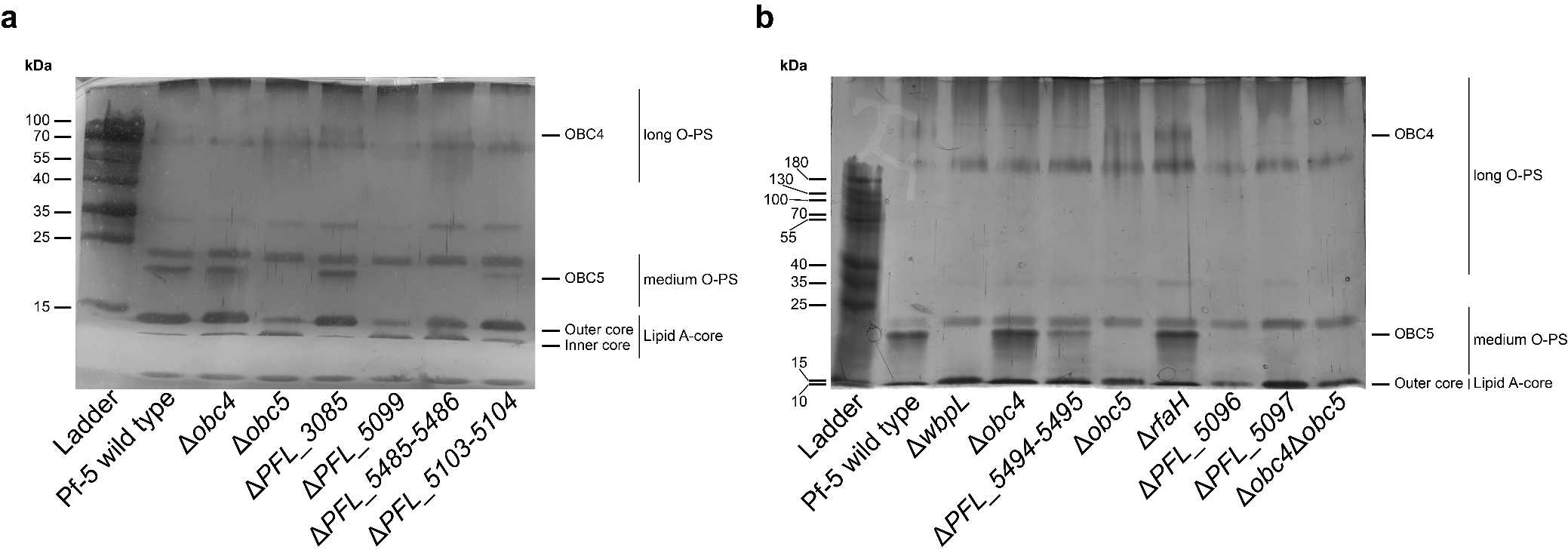


**Supplementary Figure 2. LPS profiles of P. protegens Pf-5 and mutants with deletions in the OBC4 and OBC5 gene clusters.** SDS-PAGE was performed on LPS extracted from wild type Pf-5, on Δobc4, Δobc5 and other deletion mutants associated with these O-PS clusters using 10% (**a**) and 12% acrylamide gels (**b**). To be able to clearly distinguish the long O-antigen bands, the 12% gel (**b**) was migrated for a longer time until the bands at 10 kDa reached the bottom of the gel. The percentage of acrylamide in the gels affects the migration of the LPS, explaining the differences in size when comparing them to the ladders. LPS components were visualized by silver staining. Molecular weights in kDa are depicted on the left of each gel and predicted compositions of LPS molecules (lipid A-core; O-PS, O-antigenic polysaccharide) on the right.


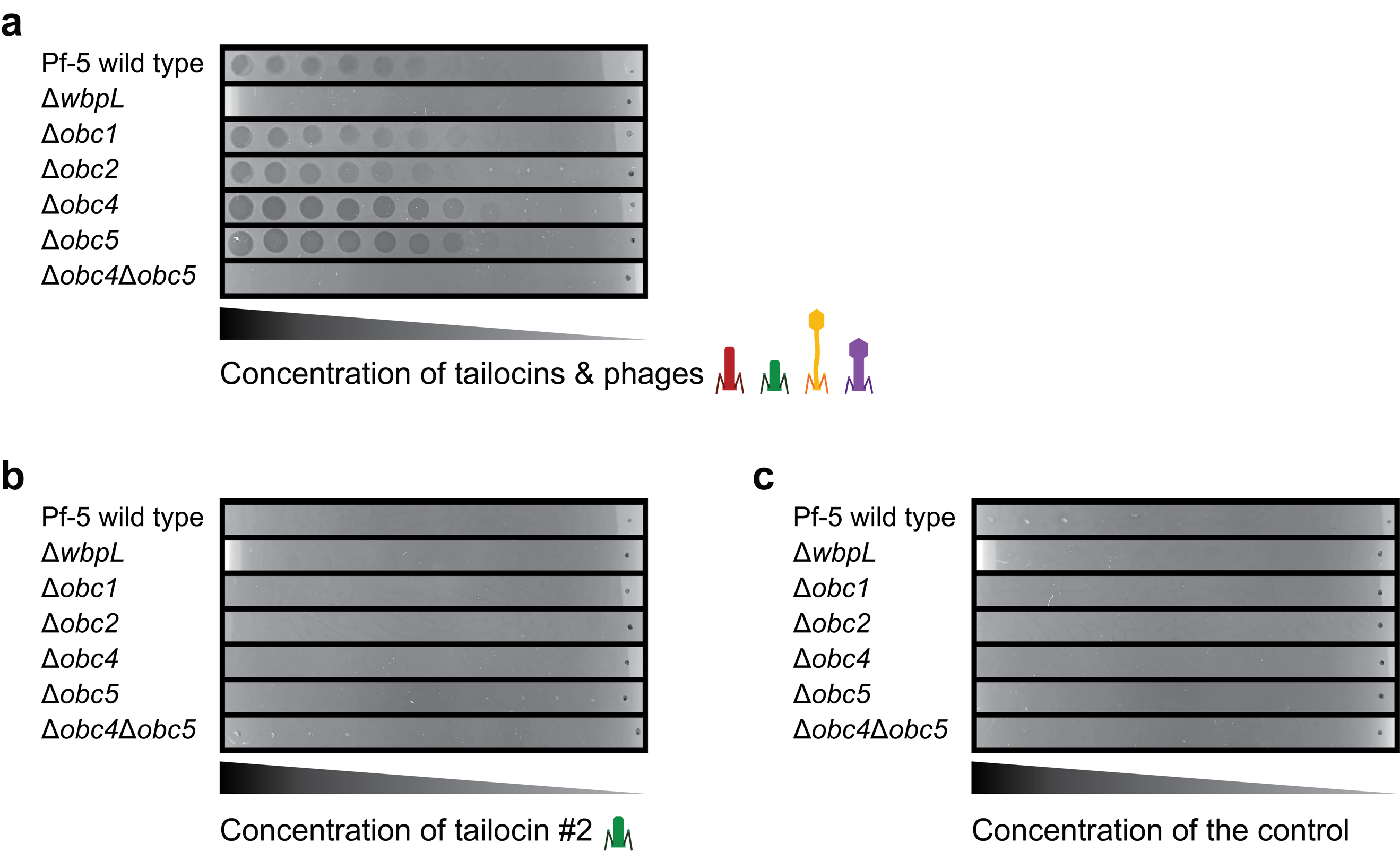


**Supplementary Figure 3. *P. protegens* Pf-5 and its LPS mutant derivatives are resistant to the tailocin #2 of CHA0**. Bacterial lawns of the different Pf-5 LPS mutants were exposed to extracts containing all tailocins and phages of CHA0 (**a**), the tailocin #2 of CHA0 (**b**) and void of any particles (control) (**c**). The different extracts were serially diluted at a ratio of 1:4.


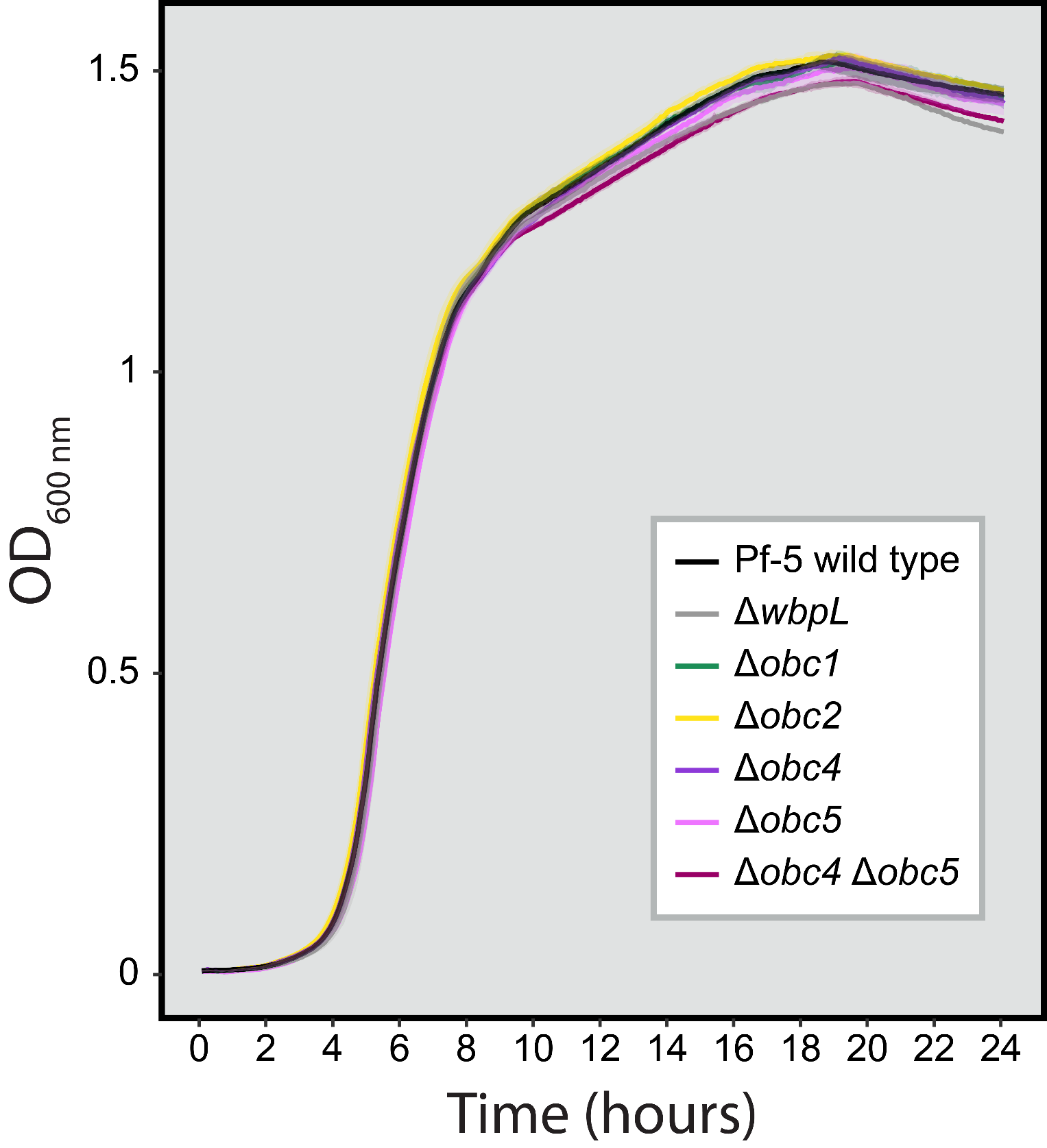


**Supplementary Figure 4*.* Growth kinetics of Pf-5 and LPS mutant derivatives.** To test for absence of eventual growth deficiencies caused by the LPS mutations, the growth of Pf-5 wild type and mutant derivatives was monitored in rich medium (NYB) for 24 h by measuring the optical density at 600 nm every 5 min in a BioTeK Synergy H1 plate reader (BioTek Instruments Inc., Winooski, VT, USA). For the growth curves, *n*=4 technical independent experiments were performed. Error-bars correspond to the standard deviation.


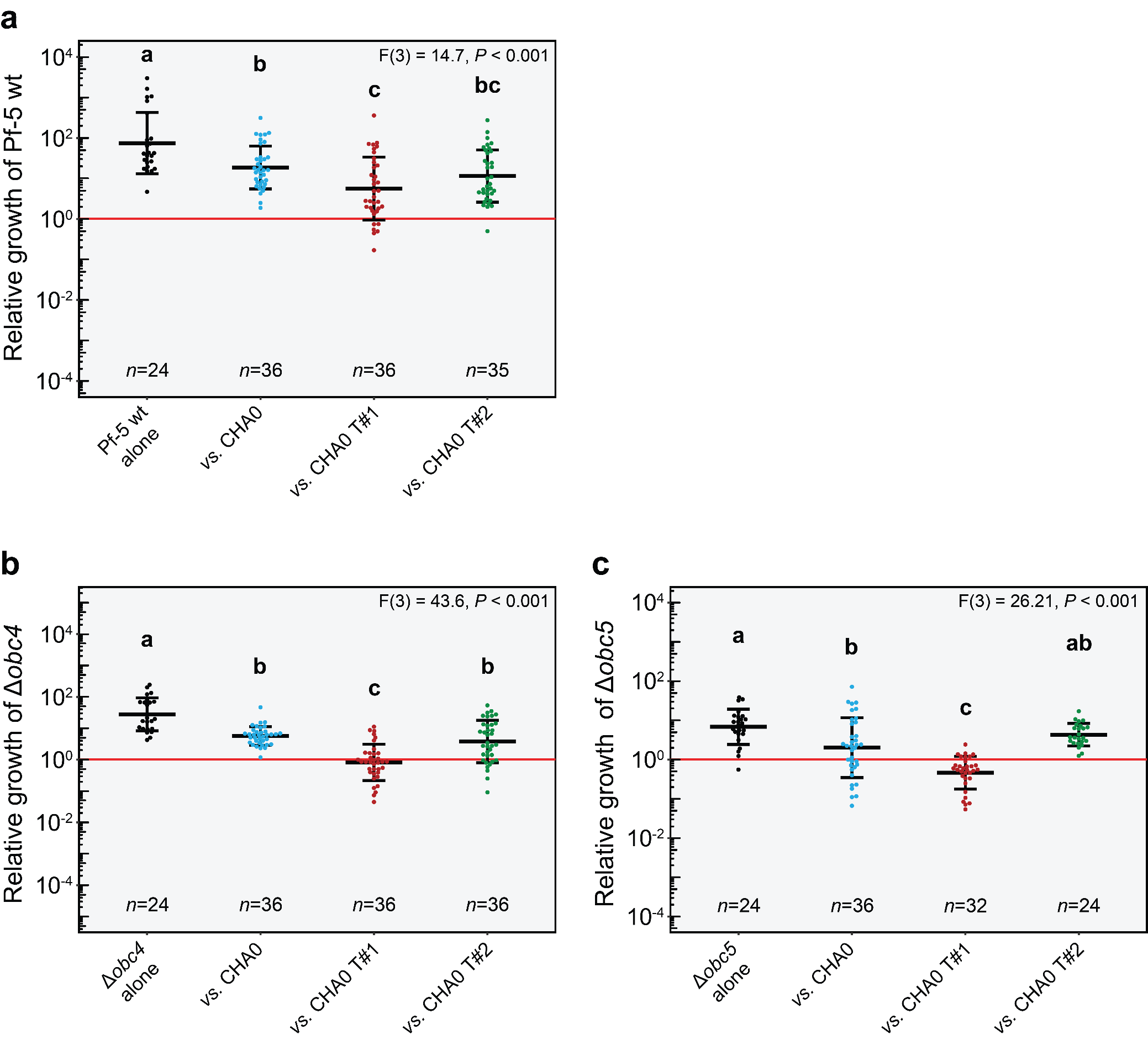


**Supplementary Figure 5. The competition with *P. protegens* CHA0 and derivatives reduces growth rates of *P. protegens* Pf-5 and derivatives *in insecta*.** The relative growth of Pf-5 and CHA0 and their mutant derivatives was assessed in *Galleria mellonella* fourth instar larvae following injection of the strains alone and in competition. The relative growth of Pf-5 wild-type (**a**) and LPS mutant derivatives, Δ*obc4* (**b**) and Δ*obc5* (**c**) was assessed alone (black) and in 1:1 ratio mixtures with CHA0 (blue) and derivatives (Δtail2ΔmyoΔsiph producing exclusively the tailocin #1, CHA0 T#1, red; Δtail1ΔmyoΔsiph producing exclusively the tailocin #2, CHA0 T#2, green) in the larvae. The red line indicates no growth after 24 h in the insect. Statistical differences were assessed by ANOVA with a Bonferroni correction and are indicated with letters a, b, c and d. Six biological replicates with each six technical replicates were performed, thus, 36 larvae were injected in total. The horizontal lines indicate the interquartile range with the center representing the median.


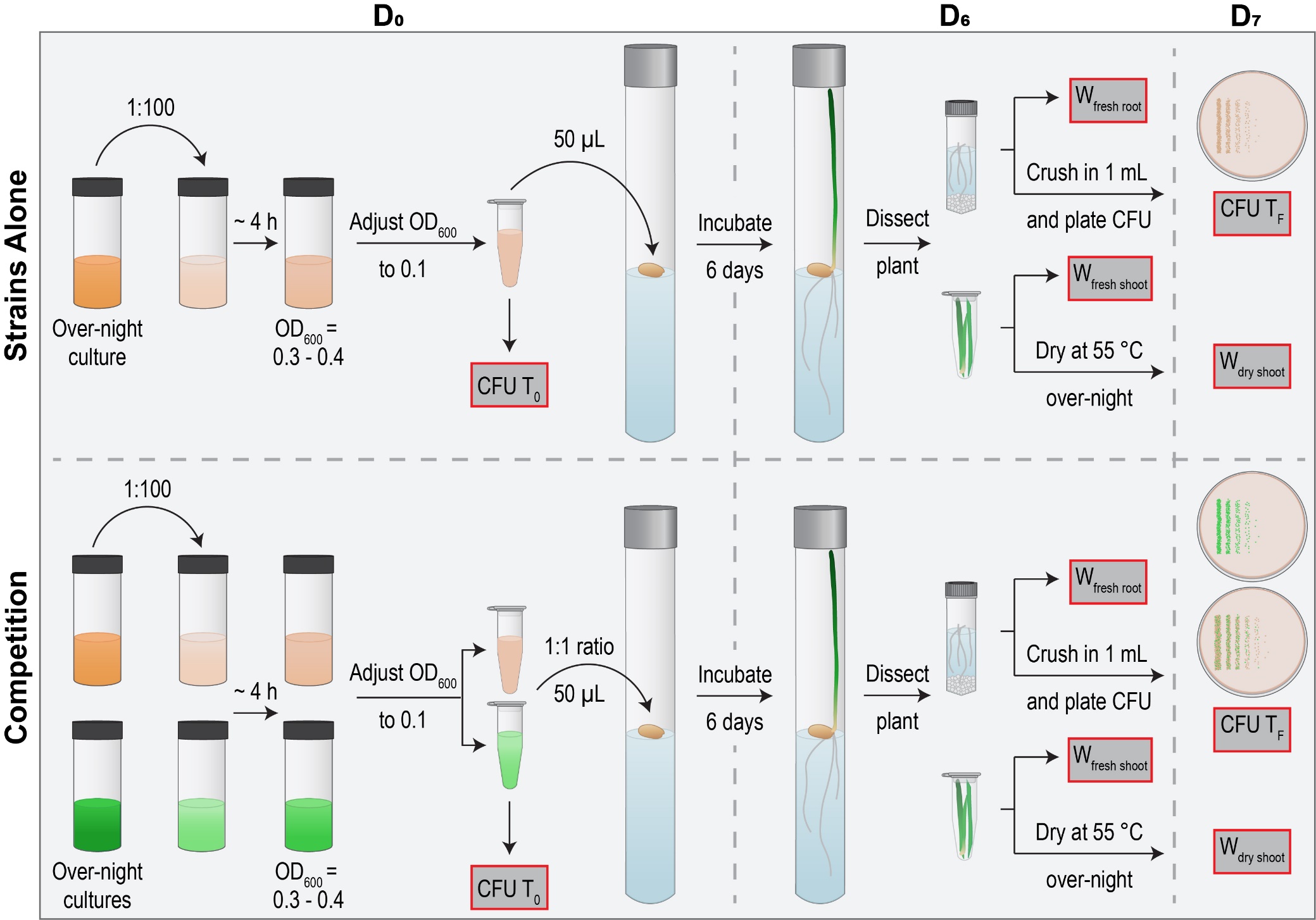


**Supplementary Figure 6. Scheme of the plant inoculation experiment.** After germinating Arina wheat cultivar seeds overnight, they were inoculated with the different bacterial cultures. We used CHA0 wild type, CHA0 T#1 and CHA0 T#2 as the CHA0 derivatives and wild type Pf-5, Δ*obc4*, Δ*obc5* and Δ*obc4*Δ*obc5* as the Pf-5 derivatives. Firstly, to assess the colonization ability of the different strains, we placed each strain alone on the seedlings. Secondly, we placed a 1:1 ratio competition mix of pairs of CHA0 derivatives *vs.* Pf-5 derivatives of interest. We used exponential growth phase cultures adjusted to an optical density of 0.1. After six days of incubation, bacteria were collected from the roots to perform CFU counting and to assess plant weights.


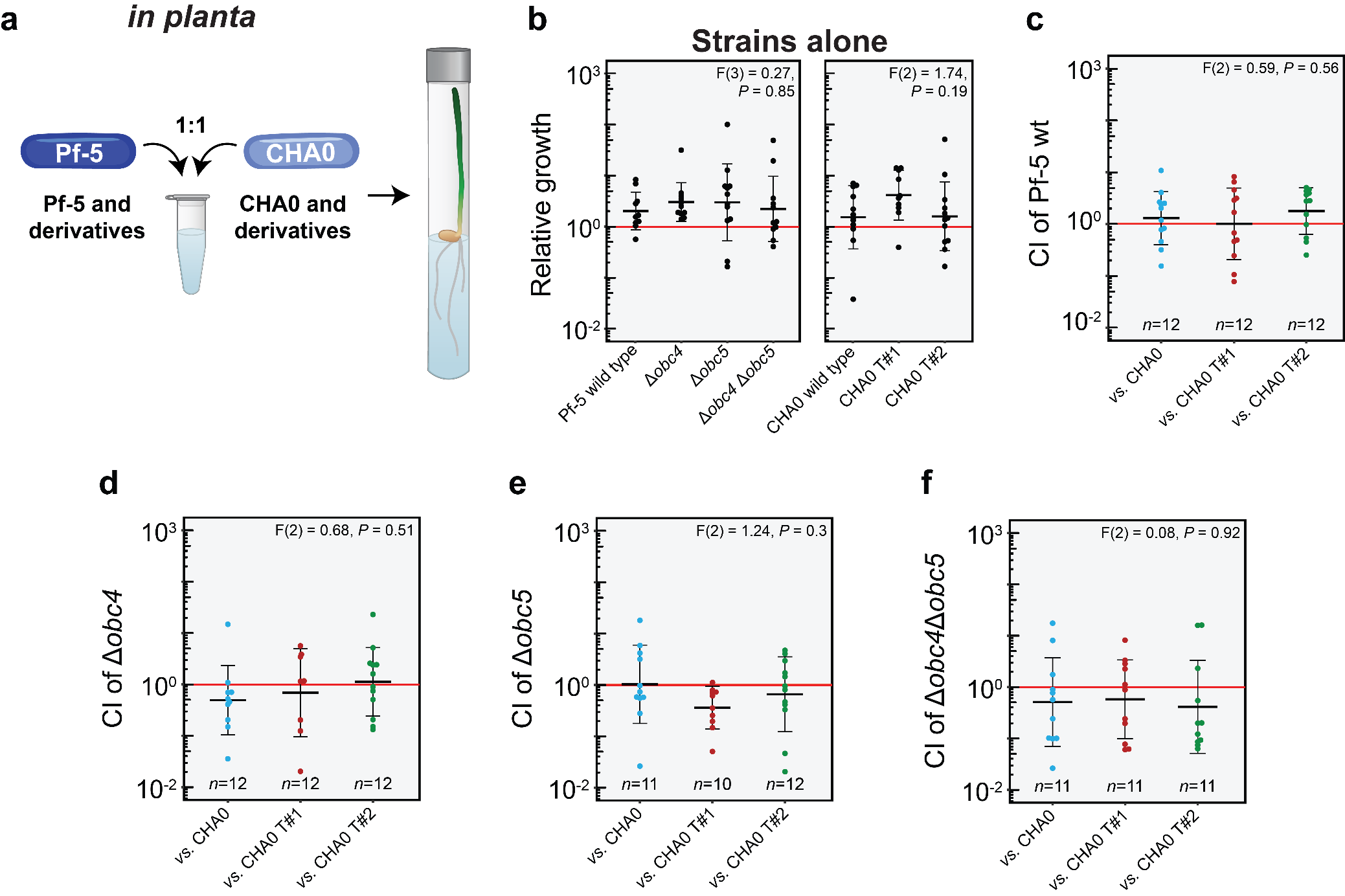


**Supplementary Figure 7. The deletion of either OBC4 or/and OBC5 does not significantly affect the competitiveness of P. protegens Pf-5 in planta.** The relative growth of Pf-5 and CHA0 and their mutant derivatives was assessed on roots of six-day-old wheat plants (**a**) with strains inoculated alone (**b**). The competitive indices (CI) of Pf-5 (**c**) and LPS mutant derivatives Δobc4 (**d**), Δobc5 (**e**), Δobc4Δobc5 (**f**) were assessed in 1:1 ratio mixtures with CHA0 (blue) and derivatives (Δtail2ΔmyoΔsiph producing exclusively the tailocin #1, CHA0 T#1 (red); Δtail1ΔmyoΔsiph producing exclusively the tailocin #2, CHA0 T#2 (green)) on the wheat roots. The red line indicates a competition where both strains would not be influenced by the presence of one another. Statistical differences were assessed by Kruskal-Wallis test using a Bonferroni correction and indicated with letters a and b. Four biological independent experiments were performed.


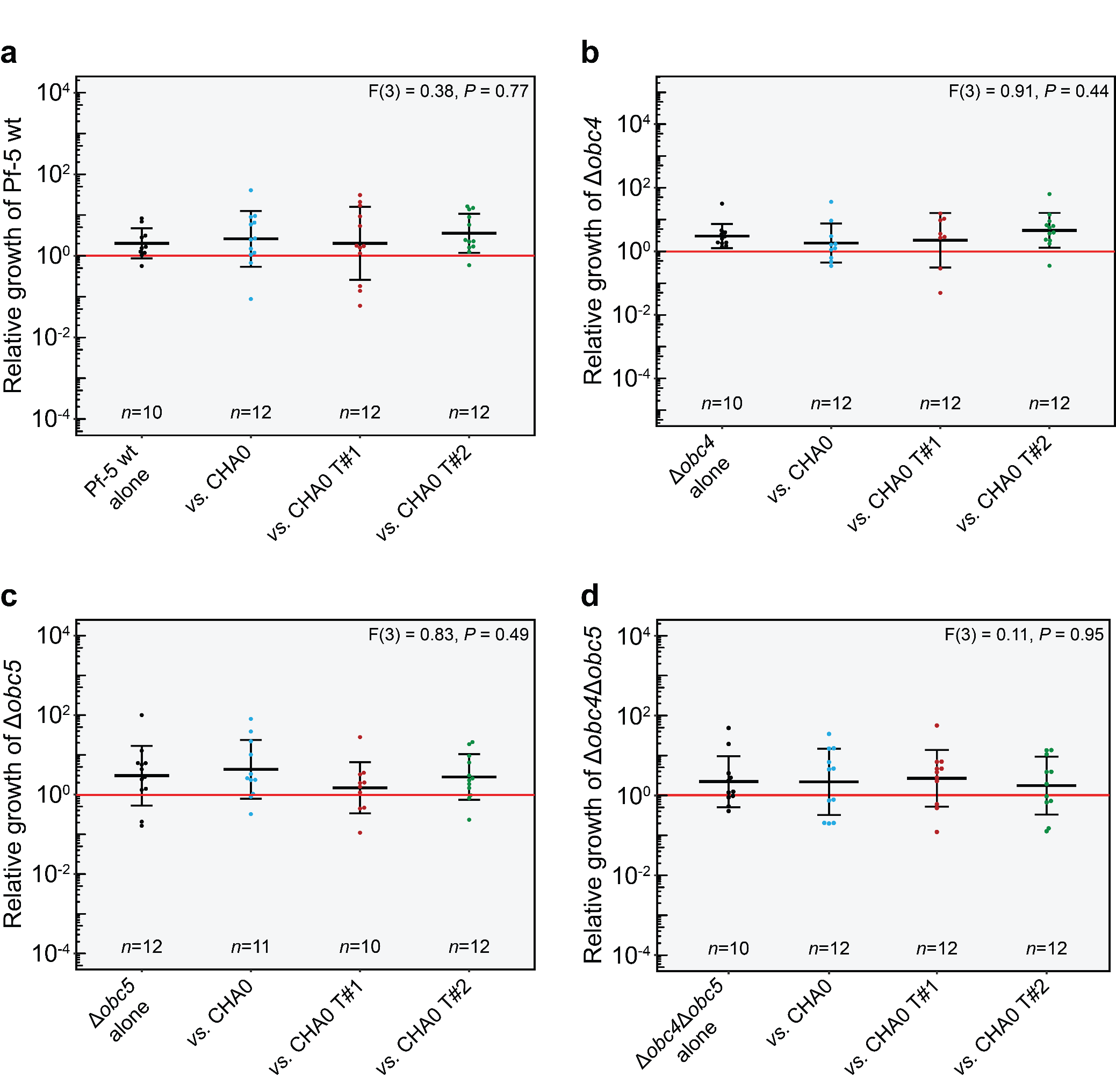


**Supplementary Figure 8. The competition with *P. protegens* CHA0 and derivatives has no impact on growth rates of *P. protegens* Pf-5 and derivatives during plant colonization.** The relative growth of Pf-5 and CHA0 and their mutant derivatives was assessed on wheat roots following inoculation of the strains alone and in competition. The relative growth of Pf-5 wild-type (**a**) and LPS mutant derivatives, Δ*obc4* (**b**), Δ*obc5* (**c**) and Δ*obc4*Δ*obc5* (**c**) was assessed alone (black) and in 1:1 ratio mixtures with CHA0 (blue) and derivatives (Δtail2ΔmyoΔsiph producing exclusively the tailocin #1, CHA0 T#1, red; Δtail1ΔmyoΔsiph producing exclusively the tailocin #2, CHA0 T#2, green) *in planta*. The red line indicates no growth after 6 days on the roots. Statistical differences were assessed by ANOVA with a Bonferroni correction and are indicated with letters a, b, c and d. Four biological independent experiments were performed. The horizontal lines indicate the interquartile range with the center representing the median.

**Supplementary Tables**

**Supplementary Table 1.** Bacterial strains used in this study.

| **Strain** ^1^ | **Genome accession no.** | **Origin** | | **Reference**  **or source** | | |  |
| --- | --- | --- | --- | --- | --- | --- | --- |
| ***Pseudomonas chlororaphis* subgroup** | | |  | |  | | |
| *P. chlororaphis* O6 | | NZ_CM001490.1 | Soil | | [9] | |  |
| *P. chlororaphis* R47 | | CP019399.1 | Potato rhizosphere | | [10] | |  |
| *P. chlororaphis* subsp. *aureofaciens* 30-84 | | NZ_CM001559.1 | Wheat rhizosphere | | [9, 11] | |  |
| *P. chlororaphis* subsp. *aurantiaca* JD37 | | NZ_CP009290.1 | Potato rhizosphere | | [12, 13] | |  |
| *P. chlororaphis* subsp. *aureofaciens* CD | | NZ_LHVB00000000.1 | Cyclops (water) | | [14, 15] | |  |
| *P. chlororaphis* subsp. *aureofaciens* LMG1245^T^ | | NZ_LHVA00000000.1 | River clay | | [15, 16] | |  |
| *P. chlororaphis* subsp. *chlororaphis* LMG5004^T^ | | NZ_LHVC00000000.1 | Contaminated plate | | [15, 17] | |  |
| *P. chlororaphis* subsp*. piscium* DSM21509^T^ (JF3835^T^) | | NZ_CP027707.1 | European perch intestine | | [18] | |  |
| *P. chlororaphis* subsp. *piscium* PCL1391 | | NZ_CP027736.1 | Tomato root | | [15, 19] | |  |
| ***Pseudomonas protegens* subgroup** | | |  | |  | |  |
| *P. protegens* BRIP | | NZ_LHUW00000000.1 | Cyclops | | [14, 15] | |  |
| *P. protegens* Cab57 | | NZ_AP014522.1 | Shepperd’s purse rhizosphere | | [20] | |  |
| *P. protegens* CHA0^T^ | | LS999205.1 | Tobacco rhizosphere | | [21, 22] | |  |
| *P. protegens* K94.41 | | NZ_LHUU00000000.1 | Cucumber rhizosphere | | [15, 23] | |  |
| *P. protegens* PF | | NZ_LHUX00000000.1 | Wheat leaves | | [15, 24] | |  |
| *P. protegens* Pf1 | | ERS3935804 | Tobacco | | [5, 24] | |  |
| *P. protegens* Pf-5 | | CP000076.1 | Cotton rhizosphere | | [25, 26] | |  |
| *P. protegens* PGNR1 | | NZ_LHUV00000000.1 | Tobacco rhizosphere | | [15, 24] | |  |
| *Pseudomonas* sp. AU11706 | | NZ_LCZB00000000.1 | Cystic fibrosis sputum | | [27] | |  |
| *Pseudomonas* sp. AU20219 | | NZ_LDET00000000.1 | Cystic fibrosis sputum | | [27] | |  |
| *Pseudomonas* sp. AU13852 | | NZ_LCZC00000000.1 | Cystic fibrosis sputum | | [27] | |  |
| *Pseudomonas* sp. CMR5c | | NZ_LHUY00000000.1 | Red cocoyam rhizosphere | | [15, 28] | |  |
| *Pseudomonas* sp. CMR12a | | CP027706.1 | Red cocoyam rhizosphere | | [15, 28] | |  |
| *Pseudomonas* sp. LD120 | | ERR3588830 | Blade of marine alga | | [29, 30] | |  |
| *Pseudomonas* sp. Os17 | | NZ_AP014627.1 | Rice rhizosphere | | [20] | |  |
| *Pseudomonas* sp. St29 | | NZ_AP014628.1 | Potato rhizosphere | | [20] | |  |

^1^ ^T^, type strain

| **Supplementary Table 2**. CHA0 derivatives, Pf-5 derivatives and *Escherichia coli* strains used in this study. | | | |
| --- | --- | --- | --- |
| **Strain name** | **Strain code** | **Genotype or relevant characteristics**^1^ | **Reference or source** |
| ***Pseudomonas protegens* CHA0 and derivatives** | | |  |
| CHA0 | CHA0^T^ | *P. protegens* type strain; wild type; genome accession no. LS999205.1 | [21, 22] |
| CHA0-*gfp2* | CHA0*-gfp2* | CHA0::attTn7-*gfp2*; Gm^R^ | [31] |
| ΔtailclusterΔsiphΔmyo | CHA5300 | CHA0 with the deletion of the entire R-tailocin cluster and of the *Myoviridae* and *Siphoviridae* prophages | [5] |
| ΔtailclusterΔsiphΔmyo*-gfp2* | CHA5300*-gfp2* | CHA0 ΔtailclusterΔsiphΔmyo::attTn7-*gfp2*; Gm^R^ | [5] |
| Δtail1ΔmyoΔsiph | CHA5301 | CHA0 with the deletion of the tailocin #1 cluster and of the *Myoviridae* and *Siphoviridae* prophages | [5] |
| Δtail1ΔmyoΔsiph*-gfp2* | CHA5301*-gfp2* | CHA0 Δtail1ΔmyoΔsiph::attTn7-*gfp2*; Gm^R^ | [5] |
| Δtail2ΔmyoΔsiph | CHA5302 | CHA0 with the deletion of the tailocin #2 cluster and of the *Myoviridae* and *Siphoviridae* prophages | [5] |
| Δtail2ΔmyoΔsiph*-gfp2* | CHA5302*-gfp2* | CHA0 Δtail2ΔmyoΔsiph::attTn7-*gfp2*; Gm^R^ | [5] |
| CHA0 Δ*wbpL* | CHA5161 | CHA0 with the deletion of PPRCHA0_4350 | [8] |
| CHA0 Δ*wzx* | CHA5206 | CHA0 with the deletion of PPRCHA0_4354 | [8] |
| CHA0 Δ*obc1* | CHA5163 | CHA0 with the deletion of the entire OBC1 cluster (PPRCHA0_2061 to 2069) | [8] |
| CHA0 Δ*obc2* | CHA5163 | CHA0 with the deletion of the entire OBC2 cluster (PPRCHA0_3086 to 3101) | [8] |
| CHA0 Δ*obc3* | CHA5182 | CHA0 with the deletion of the entire OBC3 cluster (PPRCHA0_1950 to 1958) | [8] |
| ***Pseudomonas protegens* Pf-5 and derivatives** | | |  |
| Pf-5 | Pf-5 | Wild type; genome accession no. CP000076.1 | [25, 26] |
| Pf-5 Δ*PFL_0524* | Pf5_0004 | Pf-5 with the deletion of PFL_0524 | This study |
| Pf-5 Δ*wbpL* | Pf5_0001 | Pf-5 with the deletion of *wbpL* (PFL_4306) | This study |
| Pf-5 Δ*obc1* | Pf5_0005 | Pf-5 with the deletion of the entire OBC1 cluster (PFL_2024-PFL_2031) | This study |
| Pf-5 Δ*obc2* | Pf5_0009 | Pf-5 with the deletion of the entire OBC2 cluster (PFL_3078-PFL_3089) | This study |
| Pf-5 Δ*PFL_3085* | Pf5_0010 | Pf-5 with the deletion of PFL_3085 | This study |
| Pf-5 Δ*obc4* | Pf5_0006 | Pf-5 with the deletion of the entire OBC4 cluster (PFL_5483-PFL_5496) | This study |
| Pf-5 Δ*PFL_5485-5486* | Pf5_0008 | Pf-5 with the deletion of PFL_5485-PFL_5486 | This study |
| Pf-5 Δ*PFL_5494-5495* | Pf5_0014 | Pf-5 with the deletion of PFL_5494-PFL_5495 | This study |
| Pf-5 Δ*obc5* | Pf5_0012 | Pf-5 with the deletion of the entire OBC5 cluster (PFL_5092-PFL_5107) | This study |
| Pf-5 Δ*rfaH* | Pf5_0015 | Pf-5 with the deletion of *rfaH* (PFL_5092) | This study |
| Pf-5 Δ*PFL_5096* | Pf5_0016 | Pf-5 with the deletion of PFL_5096 | This study |
| Pf-5 Δ*PFL_5097* | Pf5_0017 | Pf-5 with the deletion of PFL_5097 | This study |
| Pf-5 Δ*PFL_5099* | Pf5_0002 | Pf-5 with the deletion of PFL_5099 | This study |
| Pf-5 Δ*PFL_5013-5014* | Pf5_0007 | Pf-5 with the deletion of PFL_5103-PFL_5104 | This study |
| Pf-5 Δ*obc4*Δ*obc5* | Pf5_0013 | Pf-5 with the deletion of the entire OBC4 and OBC5 clusters | This study |
| ***Escherichia coli*** | | | |
| *E. coli* S17-1/λpir |  | Laboratory strain | [32] |
| *E. coli* DH5α |  | Laboratory strain | [33] |

^1^ Gm^R^, gentamicin resistance.

**Supplementary Table 3**. Plasmids used in this study.

| **Plasmid** | **Genotype or relevant characteristics**^1^ | **Reference**  **or source** |
| --- | --- | --- |
| pBK-miniTn7-*gfp2* | pUC19-based delivery plasmid for miniTn7-*gfp2*; mob^+^; Gm^R^, Cm^R^, Ap^R^ | [34] |
| pBK-miniTn7- mTurquoise2 | pUC18T-mini-Tn7T-Gm-Pc-mTurquoise2; Gm^R^ | [35] |
| pEMG | Expression vector; *ori*R6K, *lacZα* with two flanking I-SceI sites; Km^R^, Ap^R^ | [36] |
| pME11125 | pEMG::Δ*PFL_0524*; suicide plasmid for the deletion of PFL_0524; Km^R^ | This study |
| pME11127 | pEMG::Δ*wbpL*; suicide plasmid for the deletion of PFL_4306;Km^R^ | This study |
| pME11131 | pEMG::Δ*obc1*; suicide plasmid for the deletion of the entire OBC1 gene cluster; Km^R^ | This study |
| pME11132 | pEMG::Δ*obc2*; suicide plasmid for the deletion of the entire OBC2 gene cluster; Km^R^ | This study |
| pME11126 | pEMG::Δ*PFL_3085*; suicide plasmid for the deletion of PFL_3085; Km^R^ | This study |
| pME11133 | pEMG::Δ*obc4*; suicide plasmid for the deletion of the entire OBC4 gene cluster; Km^R^ | This study |
| pME11130 | pEMG::Δ*PFL_5485-5486*; suicide plasmid for the deletion of PFL_5485 and PFL_5486; Km^R^ | This study |
| pME11142 | pEMG::Δ*PFL_5494-5495*; suicide plasmid for the deletion of PFL_5494 and PFL_5495; Km^R^ | This study |
| pME11134 | pEMG::Δ*obc5*; suicide plasmid for the deletion of the entire OBC5 gene cluster; Km^R^ | This study |
| pME11143 | pEMG::Δ*rfaH*; suicide plasmid for the deletion of PFL_5092; Km^R^ | This study |
| pME11144 | pEMG::Δ*PFL_5096*; suicide plasmid for the deletion of PFL_5096; Km^R^ | This study |
| pME11145 | pEMG::Δ*PFL_5097*; suicide plasmid for the deletion of PFL_5097; Km^R^ | This study |
| pME11128 | pEMG::Δ*PFL_5099*; suicide plasmid for the deletion of PFL_5099; Km^R^ | This study |
| pME11129 | pEMG::ΔPFL_5013-5014; suicide plasmid for the deletion of PFL_5013 and PFL_5014; Km^R^ | This study |

^1^ Ap^R^, ampicillin resistance; Cm^R^, chloramphenicol resistance; Gm^R^, gentamycin resistance; Km^R^, kanamycin resistance.

**Supplementary Table 4**. Oligonucleotides used for the construction of the Pf-5 LPS derivative mutants.

| **Primer** | **Sequence (5’-3’)** ^1^ | | **Application** | |
| --- | --- | --- | --- | --- |
| Check PFL_0304F | | GCGTACCGCTACCCATTG | | Check of Δ*PFL_0304* |
| Check PFL_0304R | | CACAACGAGCAGAAGAAC | | Check of Δ*PFL_0304* |
| del-PFL_0524-1 | | CGGAATTCTATCTGCTGCAGGCCTTT | | Deletion of PFL_0524 |
| del-PFL_0524-2 | | GGGGTACCCATTACTGCCTCTTGCCC | | Deletion of PFL_0524 |
| del-PFL_0524-3 | | GGGGTACCAAAGACTGAAGAGACGCC | | Deletion of PFL_0524 |
| del-PFL_0524-4 | | CGGGATCCGCCGTCAATATCATCGCT | | Deletion of PFL_0524 |
| Check PFL_0524F | | TTGCTTGAAGGCATGAGC | | Check of ΔPFL_0524 |
| Check PFL_0524R | | TTCGGCAATCGAGCAGTA | | Check of ΔPFL_0524 |
| del-PFL_4306-1 | | CGGAATTCAAAGTACTGACGCATCGC | | Deletion of *wbpL* (PFL_4306) |
| del-PFL_4306-2 | | GGGGTACCGAGTTGGAGAAAACAGACTGA | | Deletion of *wbpL* (PFL_4306) |
| del-PFL_4306-3 | | GGGGTACCCACCATGTAACTTTAATCCTT | | Deletion of *wbpL* (PFL_4306) |
| del-PFL_4306-4 | | CGGGATCCCTCATTGCCTCTCAAACG | | Deletion of *wbpL* (PFL_4306) |
| check-PFL_4306F | | CATCCATAGAGCGACCCA | | Check of Δ*wbpL* |
| check-PFL_4306F | | TGCGGGTCTCTTCAGGTG | | Check of Δ*wbpL* |
| del-obc1-1 | | CGGAATTCCGAATTGGGGCGATGATA | | Deletion of the OBC1 cluster (PFL_2024-PFL_2031) |
| del-obc1-2 | | GGGGTACCTAGACCGGCAATGGCTCA | | Deletion of the OBC1 cluster (PFL_2024-PFL_2031) |
| del-obc1-3 | | GGGGTACCCAGTTCAGAGCACATCGG | | Deletion of the OBC1 cluster (PFL_2024-PFL_2031) |
| del-obc1-4 | | CGGGATCCGACTCGAACACCCAGTTC | | Deletion of the OBC1 cluster (PFL_2024-PFL_2031) |
| Check-obc1-F | | ATTTGCCCAATCATTGCT | | Check of Δ*obc1* |
| Check-obc1-R | | GATGTTGGCCAGGTCATC | | Check of Δ*obc1* |
| del-obc2-1 | | CGGAATTCTGCCAGCCATATCTTTGA | | Deletion of the OBC2 cluster (PFL_3078-PFL_3089) |
| del-obc2-2 | | CGGGATCCCAAGGGAACGACCTCGTC | | Deletion of the OBC2 cluster (PFL_3078-PFL_3089) |
| del-obc2-3 | | CGGGATCCCAATGAAACCCTGTTCCC | | Deletion of the OBC2 cluster (PFL_3078-PFL_3089) |
| del-obc2-4 | | ACGGTCGACGCTGACCCCACCGTAATA | | Deletion of the OBC2 cluster (PFL_3078-PFL_3089) |
| Check-obc2-F | | ATGCCGTAGCAGGTAATG | | Check of Δ*obc2* |
| Check-obc2-R | | AAAGCTGTAGCCTGGTAC | | Check of Δ*obc2* |
| del-PFL_3085-1 | | CGGAATTCCCGGACCTGTTTCTCGAT | | Deletion of PFL_3085 |
| del-PFL_3085-2 | | GGGGTACCCATACTCGGGAGCTCTCT | | Deletion of PFL_3085 |
| del-PFL_3085-3 | | GGGGTACCTGACAACGCCGCGTG | | Deletion of PFL_3085 |
| del-PFL_3085-4 | | CGGGATCCGACCCGTTCGAACCA | | Deletion of PFL_3085 |
| Check-PFL_3085-F | | CTGAGTTCACGCACCGAA | | Check of Δ*PFL_3085* |
| Check-PFL_3085-R | | CACCACTATCAGTTCGCA | | Check of Δ*PFL_3085* |
| del-obc4-1 | | CGGAATTCGGCAAGGTCATCTGGTTT | | Deletion of the OBC4 cluster (PFL_5483-PFL_5496) |
| del-obc4-2 | | GGGGTACCTAGTTCCAGCACTTGCTG | | Deletion of the OBC4 cluster (PFL_5483-PFL_5496) |
| del-obc4-3 | | GGGGTACCCATTTACCCGCCTTGACT | | Deletion of the OBC4 cluster (PFL_5483-PFL_5496) |
| del-obc4-4 | | CGGGATCCCGAGCAAGGTGCTTTGAT | | Deletion of the OBC4 cluster (PFL_5483-PFL_5496) |
| Check-obc4-F | | GATGCCGGCCTAATTGTG | | Check of Δ*obc4* |
| Check-obc4-R | | CTTGGCAGGTGAAGCATG | | Check of Δ*obc4* |
| del-PFL-5485-5486-1 | | CGGAATTCCAGGTCGTCATCGCTACC | | Deletion of PFL_5485a and PFL_5486 |
| del-PFL-5485-5486-2 | | CGGGATCCCGGCGAGGAGCATTTTAG | | Deletion of PFL_5485 and PFL_5486 |
| del-PFL-5485-5486-3 | | CGGGATCCACGCACGAATTGCATCAG | | Deletion of PFL_5485 and PFL_5486 |
| del-PFL-5485-5486-4 | | ACGGTCGACGGAGTCTTTCCGATTCGT | | Deletion of PFL_5485 and PFL_5486 |
| check-PFL-5485-5486-F | | ATTGGACAACCCTCCAGG | | Check of Δ*PFL_5485-5486* |
| check-PFL-5485-5486-R | | CGGATGTTCCGTACTGAG | | Check of Δ*PFL_5485-5486* |
| del-PFL_5494-5495-1 | | CGGAATTCCGTATACCCGTCCAGCAC | | Deletion of PFL_5494 and PFL_5495 |
| del-PFL_5494-5495-2 | | GGGGTACCGCGGTGGAAATGAATGCCTAA | | Deletion of PFL_5494 and PFL_5495 |
| del-PFL_5494-5495-3 | | GGGGTACCGTGTCGCATCAGGTGAAC | | Deletion of PFL_5494 and PFL_5495 |
| del-PFL_5494-5495-4 | | CGGGATCCCCATTTATGCAGGCGGAG | | Deletion of PFL_5494 and PFL_5495 |
| check_5494-5495_F | | CCGAGTACCCGATTCGAC | | Check of Δ*PFL_5494-5495* |
| check_5494-5495_R | | GGGTTTGCGGATGTCCTC | | Check of Δ*PFL_5494-5495* |
| v2.del-obc5-1 | | GGAATTCATCGACGACTATGGCTTT | | Deletion of the OBC5 cluster (PFL_5092-PFL_5107) |
| v2.del-obc5-2 | | CGGGATCCGTCTGGTTGATACGTAGCCAT | | Deletion of the OBC5 cluster (PFL_5092-PFL_5107) |
| v2.del-obc5-3 | | CGGGATCCACGGTCTGGCGTAAAACC | | Deletion of the OBC5 cluster (PFL_5092-PFL_5107) |
| v2.del-obc5-4 | | ACGCGTCGACCCTGGCGGATTGAAGGAG | | Deletion of the OBC5 cluster (PFL_5092-PFL_5107) |
| Check-obc5-F | | TTGACCCAAACCATGCCA | | Check of Δ*obc5* |
| Check-obc5-R | | TTGACCCAAACCATGCCA | | Check of Δ*obc5* |
| del-rfaH-1 | | CGGAATTCATCGACGACTATGGCTTT | | Deletion of *rfaH* (PFL_5092) |
| del-rfaH-2 | | GGGGTACCAGCCATTGAGTCAACAGT | | Deletion of *rfaH* (PFL_5092) |
| del-rfaH-3 | | GGGGTACCATTCGCAAGATCTAGGCC | | Deletion of *rfaH* (PFL_5092) |
| del-rfaH-4 | | CGGGATCCGATATCGCTTGAAGGGGC | | Deletion of *rfaH* (PFL_5092) |
| check-rfaH-F | | GAAGGTTTCGCGGATGAG | | Check of Δ*rfaH* |
| check-rfaH-R | | CTCTGCAGCGAAGCTTTT | | Check of Δ*rfaH* |
| del-PFL_5096-1 | | CGGAATTCGATTGGGCAGAGGATCGT | | Deletion of PFL_5096 |
| del-PFL_5096-2 | | CGGGATCCGGGAGTCATGAACGTTCC | | Deletion of PFL_5096 |
| del-PFL_5096-3 | | CGGGATCCTTCTTCGGGATTGGTTTCTAA | | Deletion of PFL_5096 |
| del-PFL_5096-4 | | ACGCGTCGACCATAGCTATTGAAGGGCG | | Deletion of PFL_5096 |
| check-5096-F | | TGCTGCGATGCTTTAGGC | | Check of Δ*PFL_5096* |
| check-5096-R | | CAGCCGTGTACCAATCTT | | Check of Δ*PFL_5096* |
| del-PFL_5097-1 | | CGGAATTCTTTGGGCAGCAATACGGA | | Deletion of PFL_5097 |
| del-PFL_5097-2 | | CGGGATCCTGCATCCGAGGCTTTCAT | | Deletion of PFL_5097 |
| del-PFL_5097-3 | | CGGGATCCCCCCGACACGATGGATAG | | Deletion of PFL_5097 |
| del-PFL_5097-4 | | ACGCGTCGACAGCTTGCAGAGTAGCTCT | | Deletion of PFL_5097 |
| check-5097-F | | GCTGTCAACAGTTCAGGC | | Check of Δ*PFL_5097* |
| check-5097-R | | AGCAGGTATTGCAAACCC | | Check of Δ*PFL_5097* |
| del-PFL_5099-1 | | CGGAATTCGATTGGTATGCCATTGCC | | Deletion of PFL_5099 |
| del-PFL_5099-2 | | GGGGTACCGAGGCTCTTCAGGTTCAT | | Deletion of PFL_5099 |
| del-PFL_5099-3 | | GGGGTACCCACTCATGAGTGCCCCAA | | Deletion of PFL_5099 |
| del-PFL_5099-4 | | CGGGATCCGGCCCAATGAGCATTGAA | | Deletion of PFL_5099 |
| check-5099-F | | CGAGACTTCATCACTCCC | | Check of Δ*PFL_5099* |
| check-5099-R | | GTAGGTTGCCTCGTTCTT | | Check of Δ*PFL_5099* |
| del-PFL_5103-5104-1 | | CGGAATTCTCCCACAAACAAGCAGTG | | Deletion of PFL_5103 and PFL_5104 |
| del-PFL_5103-5104-2 | | GGGGTACCTTTGTTTTTTACATCAGGTCTCAT | | Deletion of PFL_5103 and PFL_5104 |
| del-PFL_5103-5104-3 | | GGGGTACCAGTAAGGTTGGTTGAAATGAAAGT | | Deletion of PFL_5103 and PFL_5104 |
| del-PFL_5103-5104-4 | | CGGGATCCCAACTCTACTGTGGGCAG | | Deletion of PFL_5103 and PFL_5104 |
| check PFL_5103-5104-F | | TGGTCATACTTCACTGAAACT | | Check of Δ*PFL_5013-5014* |
| check PFL_5103-5104-R | | GATGGGCAGTGCTGTCAG | | Check of Δ*PFL_5013-5014* |
| pEMG check-F | | GTAAAACGACGGCCAGT | | Sequencing verification of the pEMG-based plasmids |
| pEMG check-R | | AACAGCTATGACCATG | | Sequencing verification of the pEMG-based plasmids |

^1^ Restriction sites are underlined**.**

**Supplementary Table 5**. Transposon sequencing characteristics.

| **Sample** | **Repetition** | **Total number of reads obtained** | **Number of cleaned reads ^a^** | **Total of HQ mapped reads ^b^** | **Percentage of HQ mapped reads ^b^** | **Mean read length (bp)** | **Total of insertion hits** | **Number of insertions every 1000 bp ^c^** | **Total genes with hits** | | **Percentage of total genes with hits** |
| --- | --- | --- | --- | --- | --- | --- | --- | --- | --- | --- | --- |
| Control | 1 | 21684055 | 11746527 | 9748737 | 82.99250493 | 111.959689 | 1035055 | 146 | 6017 | | 96.5655593 |
| Control | 2 | 18550180 | 10385088 | 8722440 | 83.99004419 | 112.247312 | 1027926 | 145 | 6017 | | 96.5655593 |
| Control | 3 | 19416863 | 11012910 | 9236150 | 83.86657114 | 112.53905 | 1037121 | 146 | 6002 | | 96.32482748 |
| Pf-5 x T#1 | 1 | 33363041 | 7582995 | 6153445 | 81.14795012 | 111.415543 | 211064 | - | 6057 | | 97.20751083 |
| Pf-5 x T#1 | 2 | 20170485 | 11680403 | 9522720 | 81.52732401 | 112.381858 | 129343 | - | 5311 | | 85.23511475 |
| Pf-5 x T#1 | 3 | 19583032 | 11045030 | 8565978 | 77.55504512 | 109.731806 | 405761 | - | 5678 | | 91.12502006 |
| ^a^ Cleaned reads: High-quality reads, i.e., trimmed reads + reads that match with the Tn5 transposon sequence. | | | | | | | | | |  |  |
| ^b^ Number of cleaned reads that were aligned to the genome of *P. protegens* Pf-5 (CP000076.1) with a mapping quality of Q20 or higher. | | | | | | | | | |  |  |
| ^c^ Total number of genes in the genome of *P. protegens* Pf-5: 6’231. | | | | | | | | | |  |  |

**References**

1. Martin M. Cutadapt removes adapter sequences from high-throughput sequencing reads. *EMBnet.journal* 2011; **17**: 10.

2. Davis MPA, van Dongen S, Abreu-Goodger C, Bartonicek N, Enright AJ. Kraken: A set of tools for quality control and analysis of high-throughput sequence data. *Methods* 2013; **63**: 41–49.

3. Li H, Durbin R. Fast and accurate short read alignment with Burrows-Wheeler transform. *Bioinformatics* 2009; **25**: 1754–1760.

4. DeJesus MA, Ambadipudi C, Baker R, Sassetti C, Ioerger TR. TRANSIT - A software tool for Himar1 TnSeq analysis. *PLoS Comput Biol* 2015; **11**: e1004401.

5. Vacheron J, Heiman CM, Keel C. Live cell dynamics of production, explosive release and killing activity of phage tail-like weapons for *Pseudomonas* kin exclusion. *Commun Biol* 2021.

6. Vacheron J, Péchy-Tarr M, Brochet S, Heiman CM, Stojiljkovic M, Maurhofer M, *et al.* T6SS contributes to gut microbiome invasion and killing of an herbivorous pest insect by plant-beneficial *Pseudomonas* *protegens*. *ISME J* 2019; 1.

7. Rochat L, Péchy-Tarr M, Baehler E, Maurhofer M, Keel C. Combination of fluorescent reporters for simultaneous monitoring of root colonization and antifungal gene expression by a biocontrol pseudomonad on cereals with flow cytometry. *Mol Plant Microbe Interact* 2010; **23**: 949–961.

8. Kupferschmied P, Chai T, Flury P, Blom J, Smits THM, Maurhofer M, *et al.* Specific surface glycan decorations enable antimicrobial peptide resistance in plant-beneficial pseudomonads with insect-pathogenic properties. *Environ Microbiol* 2016; **18**: 4265–4281.

9. Loper JE, Hassan KA, Mavrodi D V., Davis EW, Lim CK, Shaffer BT, *et al.* Comparative genomics of plant-associated *Pseudomonas* spp.: insights into diversity and inheritance of traits involved in multitrophic interactions. *PLoS Genet* 2012; **8**: e1002784.

10. De Vrieze M, Pandey P, Bucheli TD, Varadarajan AR, Ahrens CH, Weisskopf L, *et al.* Volatile organic compounds from native potato-associated *Pseudomonas* as potential anti-oomycete agents. *Front Microbiol* 2015; **6**: 1295.

11. Pierson LS, Thomashow LS. Cloning and heterologous expression of the phenazine biosynthetic locus from *Pseudomonas aureofaciens* 30-84. *Mol Plant Microbe Interact* 1992; **5**: 330–9.

12. Jiang Q, Xiao J, Zhou C, Mu Y, Xu B, He Q, *et al.* Complete genome sequence of the plant growth-promoting rhizobacterium *Pseudomonas aurantiaca* strain JD37. *J Biotechnol* 2014; **192**: 85–86.

13. Fang R, Lin J, Yao S, Wang Y, Wang J, Zhou C, *et al.* Promotion of plant growth, biological control and induced systemic resistance in maize by *Pseudomonas aurantiaca* JD37. *Ann Microbiol* 2013; **63**: 1177–1185.

14. Ruffner B, Péchy-Tarr M, Höfte M, Bloemberg G, Grunder J, Keel C, *et al.* Evolutionary patchwork of an insecticidal toxin shared between plant-associated pseudomonads and the insect pathogens *Photorhabdus* and *Xenorhabdus*. *BMC Genomics* 2015; **16**: 609.

15. Flury P, Aellen N, Ruffner B, Péchy-Tarr M, Fataar S, Metla Z, *et al*. Insect pathogenicity in plant-beneficial pseudomonads: phylogenetic distribution and comparative genomics. *ISME J* 2016; **10**: 2527–2542.

16. Kluyver AJ. *Pseudomonas aureofaciens* nov. spec. and its pigments. *J Bacteriol* 1956; **72**: 406–11.

17. Peix A, Valverde A, Rivas R, Igual JM, Ramirez-Bahena M-H, Mateos PF, *et al*. Reclassification of *Pseudomonas aurantiaca* as a synonym of *Pseudomonas chlororaphis* and proposal of three subspecies, *P. chlororaphis* subsp. *chlororaphis* subsp. nov., *P. chlororaphis* subsp. *aureofaciens* subsp. nov., comb. nov. and *P. chlororaphis* subsp. *aurantiaca* subsp. nov., comb. nov. *Int J Syst Evol Microbiol* 2007; **57**: 1286–1290.

18. Burr SE, Gobeli S, Kuhnert P, Goldschmidt-Clermont E, Frey J. *Pseudomonas chlororaphis* subsp. *piscium* subsp. nov., isolated from freshwater fish. *Int J Syst Evol Microbiol* 2010; **60**: 2753–2757.

19. Chin-A-Woeng TFC, Bloemberg G V., Van Der Bij AJ, Van Der Drift KMGM, Schripsema J, Kroon B, *et al*. Biocontrol by phenazine-1-carboxamide-producing *Pseudomonas chlororaphis* PCL1391 of tomato root rot caused by *Fusarium oxysporum* f. sp. radicis-lycopersici. *Mol Plant-Microbe Interact* 1998; **11**: 1069–1077.

20. Takeuchi K, Noda N, Katayose Y, Mukai Y, Numa H, Yamada K, *et al*. Rhizoxin analogs contribute to the biocontrol activity of a newly isolated *Pseudomonas* strain. *Mol Plant-Microbe Interact* 2015; **28**: 333–342.

21. Stutz EW. Naturally occurring fluorescent pseudomonads involved in suppression of black root rot of tobacco. *Phytopathology* 1986; **76**: 181.

22. Smits THM, Rezzonico F, Frasson D, Vesga P, Vacheron J, Blom J, *et al*. Updated genome sequence and annotation for the full genome of *Pseudomonas protegens* CHA0. *Microbiol Resour Announc* 2019; **8**.

23. Wang C, Ramette A, Punjasamarnwong P, Zala M, Natsch A, Moënne-Loccoz Y, *et al*. Cosmopolitan distribution of phlD-containing dicotyledonous crop-associated biocontrol pseudomonads of worldwide origin. *FEMS Microbiol Ecol* 2001; **37**: 105–116.

24. Keel C, Weller DM, Natsch A, Défago G, Cook RJ, Thomashow LS. Conservation of the 2,4-diacetylphloroglucinol biosynthesis locus among fluorescent *Pseudomonas* strains from diverse geographic locations. *Appl Environ Microbiol* 1996; **62**: 552–63.

25. Howell CR, Stipanovic RD. Control of Rhizoctonia solani on cotton seedlings with *Pseudomonas fluorescens* and with an antibiotic produced by the bacterium.

26. Paulsen IT, Press CM, Ravel J, Kobayashi DY, Myers GSA, Mavrodi D V., *et al.* Complete genome sequence of the plant commensal *Pseudomonas fluorescens* Pf-5. *Nat Biotechnol* 2005; **23**: 873–878.

27. Scales BS, Erb-Downward JR, LiPuma JJ, Huffnagle GB. Draft genome sequences of five *Pseudomonas fluorescens* subclade I and II strains, isolated from human respiratory samples. *Genome Announc* 2015; **3**: e00837-15.

28. Perneel M, Heyrman J, Adiobo A, De Maeyer K, Raaijmakers JM, De Vos P, *et al.* Characterization of CMR5c and CMR12a, novel fluorescent *Pseudomonas* strains from the cocoyam rhizosphere with biocontrol activity. *J Appl Microbiol* 2007; **103**: 1007–1020.

29. Nagel K, Schneemann I, Kajahn I, Labes A, Wiese J, Imhoff J. Beneficial effects of 2,4-diacetylphloroglucinol- producing pseudomonads on the marine alga *Saccharina latissima*. *Aquat Microb Ecol* 2012; **67**: 239–249.

30. Heiman CM, Wiese J, Kupferschmied P, Maurhofer M, Keel C, Vacheron J. Draft genome sequence of *Pseudomonas* sp. strain LD120, isolated from the marine alga *Saccharina latissima*. *Microbiol Resour Announc* 2020; **9**.

31. Péchy-Tarr M, Borel N, Kupferschmied P, Turner V, Binggeli O, Radovanovic D, *et al.* Control and host-dependent activation of insect toxin expression in a root-associated biocontrol pseudomonad. *Environ Microbiol* 2013; **15**: 736–750.

32. Simon R, Priefer U, Pühler A. A broad host range mobilization system for in vivo genetic engineering: transposon mutagenesis in gram negative bacteria. *Bio/Technology* 1983; **1**: 784–791.

33. Sambrook J, Fritsch EF, Maniatis T. Molecular cloning: a laboratory manual. *Cold Spring Harb Lab Press* . 1989. , 626

34. Koch B, Jensen LE, Nybroe O. A panel of Tn7-based vectors for insertion of the gfp marker gene or for delivery of cloned DNA into Gram-negative bacteria at a neutral chromosomal site. *J Microbiol Methods* 2001; **45**: 187–195.

35. Wilton R, Ahrendt AJ, Shinde S, Sholto-Douglas DJ, Johnson JL, Brennan MB, *et al*. A new suite of plasmid vectors for fluorescence-based imaging of root colonizing pseudomonads. *Front Plant Sci* 2018; **8**.

36. Martínez-García E, de Lorenzo V. Engineering multiple genomic deletions in Gram-negative bacteria: analysis of the multi-resistant antibiotic profile of *Pseudomonas putida* KT2440. *Environ Microbiol* 2011; **13**: 2702–2716.
